# Mutational analysis of epidermal and hyperproliferative type I keratins in mild and moderate psoriasis vulgaris patients: a possible role in the pathogenesis of psoriasis along with disease severity

**DOI:** 10.1186/s40246-018-0158-2

**Published:** 2018-05-21

**Authors:** Tamilselvi Elango, Jingying Sun, Caihong Zhu, Fusheng Zhou, Yaohua Zhang, Liangdan Sun, Sen Yang, Xuejun Zhang

**Affiliations:** 1 Institute and Department of Dermatology, The First Affiliated Hospital, Anhui Medical University, Hefei, China; 2 Key Laboratory of Dermatology, Ministry of Education, Anhui Medical University, Hefei, China; 3 Collaborative Innovation Center for Complex and Severe Dermatosis, Anhui Medical University, Hefei, China; 4 Institute of Dermatology, Huashan Hospital, Fudan University, Shanghai, China; 5 Anhui Medical University, 81 Meishan Road, Hefei, Anhui Province China

**Keywords:** Epidermal keratins, Hyperproliferative keratins, Psoriasis, Mutation, Sanger sequence, Immunofluorescence

## Abstract

**Background:**

Mutations in keratin proteins have been vastly associated with a wide array of genodermatoses; however, mutations of keratins in psoriasis have not been fully investigated. The main aim of the current research was to identify the mutation in K14, K10, K16, and K17 genes in two stages of psoriasis patients.

**Methods:**

Ninety-six psoriatic skin biopsies were collected. mRNA transcript of K14, K10, K16, and K17 was prepared, amplified, and sequenced. Sanger sequences of all keratins were further validated for mutational analysis using Mutation Surveyor and Alamut Visual. Then, in silico analysis of protein stability and protein and gene expression of all keratins was performed and validated.

**Results:**

Out of 44 mutations, about 75% of keratins are highly pathogenic and deleterious. Remaining 25% mutations are less pathogenic and tolerated in nature. In these 33 deleterious mutations were immensely found to decrease keratin protein stability. We also found a correlation between keratin and Psoriasis Area and Severity Index score which added that alteration in keratin gene in skin causes severity of psoriasis.

**Conclusions:**

We strongly concluded that acanthosis and abnormal terminal differentiation was mainly due to the mutation in epidermal keratins. In turn, disease severity and relapsing of psoriasis are mainly due to the mutation of hyperproliferative keratins. These novel keratin mutations in psoriatic epidermis might be one of the causative factors for psoriasis.

**Electronic supplementary material:**

The online version of this article (10.1186/s40246-018-0158-2) contains supplementary material, which is available to authorized users.

## Background

Keratinocytes play a special role in sensing epidermal barrier and regulating immune homeostasis [1]. Its functions mainly depend on structural proteins including keratins [2, 3]. Keratins are the main intermediate filament cytoskeleton in all epithelia [4]. In normal skin, basal cells express two keratins, K5 and K14, whereas suprabasal cells express differentiation-related keratins K1 and K10. These pairs act as a flexible scaffold enabling cells to resist physical stress and also play different cellular functions including protection from apoptosis and regulation of epithelial organization [5, 6]. Consequently, defects in intermediate filaments (Ifs) can lead to cell fragility and are linked to a wide array of genodermatoses and cancers [7, 8].

Psoriasis is a genetically determined chronic inflammatory skin disease characterized by acanthosis, abnormal keratinization, and inflammatory cell infiltrates [9]. In psoriatic lesions, terminal differentiation of keratinocytes is incomplete, which leads to preferential activation and proliferation of cells that do not mature properly. These changes are mainly associated with altered expression of keratin in psoriasis [10]. In psoriasis, the changes in keratin expression include a reduction in K1 and K10 and the induction of hyperproliferation-associated keratins K6, K16, and K17 [11–13]. Furthermore, the expression levels of K5 and K14 in the basal cell layer are also altered in the psoriatic epidermis. The hyperproliferation in psoriasis seemed to result from an increase in the number of transit amplifying cells, following depletion of the stem cell compartment [14, 15]. As a whole, these changes suggest that each keratin pair provides specific functional requirements to epidermal keratinocytes.

Over 90% of pathogenic mutations in keratinopathies are missense mutations with a small number of small in-frame insertions vs. deletion mutations and a few intronic splice site defects leading to larger in-frame deletions. Various cutaneous disorders are identified with mutated keratin proteins namely epidermolysis bullosa simplex (EBS) (K5, K14), ichthyosis (Kl, K2, and K10), palmoplantar keratoderma (K9), type I pachyonychia congenita (PC) (K6a and K16), seven type II pachyonychia congenita (K6b and K17), and monilethrix (K81, K83, and K86). [16–18]. In most of these conditions, the associated pathology results from fragile keratinocytes expressing the mutated keratin protein.

Since genome-wide association studies (GWAS), connecting the psoriasis to the late cornified envelope gene cluster has specified that epidermal abnormalities along with hyperproliferative keratin pattern play a major role in the pathogenesis of psoriasis [19, 20]. The main aim of this study is to identify the mutation of keratin in two stages of psoriasis, which mainly causes hyperproliferation along with defects in the cornification process. We speculated that mutation in K14 and K10 might serve as an important mechanism that affects keratinocyte proliferation in all stages of psoriasis, and also, mutation in hyperproliferative keratin K16 and K17 might be the foremost cause for the incurability and exacerbation of this disease. To verify our hypotheses, we examined Sanger sequence of the CDS region of all these keratins in both stages. For mutational analysis, we performed a computational analysis of all these keratins in both stages of psoriasis by using Mutation Surveyor and Alamut Visual software. In addition, effects of these mutations on protein stability were predicted by using in silico prediction tools. According to our result, the predicted deleterious mutations were mainly clustered in the rod domain on the keratin protein, which is crucial for keratin function. Most of the deleterious mutations were predicted to decrease protein stability which might cause the changes in protein expression of these keratins. All these changes might trigger or exacerbate psoriasis.

## Materials and methods

### Patients’ details

Patients with psoriasis vulgaris (*n* = 96) who visited the Institute of Dermatology, Anhui Medical University (AHMU), between 2015 and 2017 were recruited in this study. The age of all patients ranged from 16 to 71 years (mean ± SD, 37.97 ± 14.03 years). In this study, based on body surface area involvement, we have categorized patients into two, (i) mild psoriasis patients (*n* = 48) and (ii) moderate psoriasis patients (*n* = 48). Severity of plaque psoriasis was graded into mild and moderate to severe disease. Mild disease was defined as body surface area (BSA) ≤ 10, Psoriasis Area and Severity Index (PASI) ≤ 10, and dermatology life quality index (DLQI) ≤ 10 and moderate to severe psoriasis as BSA > 10 or PASI > 10 and DLQI > 10 [21]. Patients were included in the study based on the following criteria: (i) at least one well-demarcated, erythematous, scaly lesion verified by at least two dermatologists; (ii) each lesion tissue was confirmed by clinical histopathology; (iii) no systematic anti-psoriatic treatment 2 weeks before skin biopsy; and (iv) no topical anti-psoriatic treatments for 1 week prior to biopsy. Informed consent was obtained from all individuals, under an AHMU-approved protocol. The study was approved by the institutional ethical committee and conducted according to the Declaration of Helsinki principles.

### Collection of tissue samples

In all patients, 10 mm of lesional and nonlesional skin biopsies were taken after local anesthesia, lidocaine hydrochloride, and adrenaline bitartrate IP were given intradermally. Nonlesional skin biopsy served as control. Biopsies of psoriatic lesional skin were taken within a lesion, 1 cm from the edge of the plaque border. Biopsies of nonlesional skin were taken 2 cm beyond the plaque border. Skin biopsies were immediately frozen in liquid nitrogen.

### Histology analysis

Formalin-fixed skin biopsy was embedded in paraffin and processed routinely. Hematoxylin-eosin staining was used to examine the histological changes in mild and moderate psoriatic skin.

### RNA isolation and qRT-PCR

RNA from both skin tissues were isolated by TRIZOL Method, and RT-PCR was conducted using the High-Capacity cDNA Reverse Transcription Kits (Applied Biosystems, USA) according to the manufacturer’s protocol. Taqman Master Mix (Applied Biosystems, Bedford, MA) was used with Taqman probes. Real-time quantitative PCR was performed with the ABI PRISM7700 Sequence Detection System. All expression values were normalized against GAPDH. Relative mRNA expression levels of all examined genes were measured using the comparative 2^−ΔΔCT^ [22]. All amplifications were done three times in triplicate.

### PCR amplification, sequencing, and mutation screening

cDNA prepared from mild and moderate psoriatic RNA samples were amplified using a polymerase chain reaction (PCR). The primers were designed from the CDS region of all these keratins (Table 1). PCR reaction was performed using Biorad Thermal Cycler. The PCR conditions were as given in Table 1. Amplified PCR products were electrophoresed through a 1.5% agarose gel, to control the quality of fragments. Sanger sequencing reactions were performed on the purified PCR fragments using a BigDye® Terminator v3.1 Cycle Sequencing Kit (Applied Biosystems, USA) with the same primers as for the PCRs, forward and reverse primers in separate experiments. All genes were sequenced from both directions. Reactions were processed on the ABI3730xl instrument. Sequence reads were analyzed using Applied Biosystems. Sequence reads were aligned to human genome by using Bioedit software.

**Table 1:** Primer sequences and reaction conditions used for PCR

S. no.	Primer	Sequences (5′-3′)	Length (bp)	Reaction conditions
1	K14 F	GTGGGCAGTGAGAAGGTGAC	966	Denaturation, 30 s at 94 °C Annealing, 45 s at 59 °C Extension, 60 s at 72 °C No. of cycles, 35
	K14 R	AGAGGAGAACTGGGAGGAGG		
2	K10 F	GGCTCATCAGGTGGCTAT	812	Denaturation, 30 s at 94 °C Annealing, 45 s at 58 °C Extension, 60 s at 72 °C No. of cycles, 35
	K10 R	CAGGCTTCAGCATCTTTG		
3	K16 F	GTGAAGATCCGTGACTGG	856	Denaturation, 30 s at 94 °C Annealing, 45 s at 56 °C Extension,60 s at 72 °C No. of cycles, 35
	K16 R	TGCTGGGAGGAAAGGTGG		
4	K17 F	CTTCCGCACCAAGTTTGA	753	Denaturation, 30 s at 94 °C Annealing, 45 s at 55 °C Extension, 60 s at 72 °C No. of cycles, 35
	K17 R	TTGCCATCCTGGACCTCT		

Mutation Surveyor is a useful in silico tool developed by SoftGenetics that assists the detection of sequence variations within Sanger sequencing traces. This tool can process up to 400 lanes of data at a time with high accuracy and sensitivity. It can effectively detect SNPs and mutation in their homozygous or heterozygous states as well as mosaicism [23]. In this study, we did sequence analysis and pathogenic variant identification by using Mutation Surveyor DNA variant analysis software (SoftGenetics, USA). For prediction of the mutation’s pathogenicity, Alamut Visual software (Interactive Biosoftware, France) was used [24]. Studies have shown that Alamut Visual is the original decision-support software application, used by leading genetic clinicians and researchers around the world. This software was used for alignment, conservation, SIFT/PolyPhen dbSNP, and Exome Sequencing Project data collection to evaluate the variant in its surrounding genomic context [25, 26]. All variants were further annotated with the Exome Aggregation Consortium (ExAC) [27].

### Prediction of deleterious mutations

Six best performing tools were combined into a consensus classifier PredictSNP1.0, which gives significantly improved prediction performance and at the same time returned results for all mutations, confirming that consensus prediction represents an accurate and robust alternative to the predictions delivered by individual tools. Also, this is a user-friendly web interface for all researchers and clinician which enables an easy access to all eight prediction tools, the consensus classifier PredictSNP, and annotations from the Protein Mutant Database and the UniProt database. Based on the above details, we used PredictSNP1.0 (http://loschmidt.chemi.muni.cz/predictsnp1), classifier webserver, to predict the effects of mutations on protein function [28]. PredictSNP1.0 runs and integrates MAPP, nsSNPAnalyzer, PANTHER, PhD-SNP, Polyphen-1, Polyphen-2, SIFT, and SNAP to predict disease-related amino acid mutations. The four tools use machine-learning methods; nsSNPAnalyzer uses random forest, PhD-SNP uses support vector machines, PolyPhen-2 uses Naive Bayes, and SNAP uses neural network. SIFT, MAPP, and PANTHER consider alignment score information, and PolyPhen-1 uses an expert set of empirical rules to predict possible impact of amino acid substitutions. PredictSNP1.0 displays the confidence scores generated by each tool and a consensus prediction as percentages by using their observed accuracy values to ease comparisons [29].

### The effects of predicted deleterious mutations on protein stability

The effect of deleterious missense amino acid substitutions on K14, K10, K16, and K17 protein stability was analyzed with MUpro (http://mupro.proteomics.ics.uci.edu) [30] and I-Mutant2.0 (http://folding.biofold.org/i-mutant/i-mutant2.0.html) [31]. MUpro uses support vector machine (SVM) and neural network to predict the effect of amino acid changes on protein stability and calculates a score between − 1 and 1 as the confidence of prediction. A confidence score < 0 indicates the mutation decreases the protein stability, while a confidence score > 0 means the mutation increases the protein stability. I-Mutant 2.0 uses SVM to predict protein stability alterations upon mutations and provides the predicted free energy change value (DDG) and the sign of the prediction as increase or decrease. DDG is calculated as unfolding Gibbs free energy value (mutated protein) − unfolding Gibbs free energy value (wild-type protein) in Kcal/mole. DDG > 0 means increased protein stability, and DDG < 0 means decreased protein stability.

### Visualization of predicted deleterious mutations on 3D keratin structure

The location of predicted deleterious mutations was detected by STRUM (http://zhanglab.ccmb.med.umich.edu/STRUM/), which constructs 3D models according to the iterative threading assembly refinement (I-TASSER) simulation [32].

### Western blot analysis

Western blot was performed as previously described [33] using primary antibodies as follows: K14, K10, K16, and K17 and β-actin (Thermo-scientific, USA). Band intensity was analyzed using ImageJ software (http://rsb.info.nih.gov/ij/).

### Immunofluorescence and confocal microscopy

Immunofluorescence assay was performed as described previously [34]. Skin biopsies were obtained from the lesional and nonlesional skin of each patient (control samples). Tissues were sectioned (4 μm) and stained with rabbit monoclonal anti-cytokeratin 14 and mouse monoclonal anti-cytokeratin10, 16, and 17 (Thermo-scientific, USA). An appropriate Alexa Fluor 488-conjugated anti-rabbit IgG and anti-mouse IgG (Thermo-scientific, USA) and DAPI staining of nuclei were used. The images were examined by the pathologist using an Olympus–Ix Microscope and merged using Image J Software.

### Statistical analysis

Results were expressed as mean ± SD. Data were analyzed using SPSS 16.0 software. Nonlesional and lesional skin biopsy samples were compared by Student’s *T* test. Spearman’s coefficient was used to identify correlations between keratins and PASI score. *P* < 0.05 was considered to be significant.

## Results

### Baseline analysis of psoriatic lesion

**Fig. 1:**
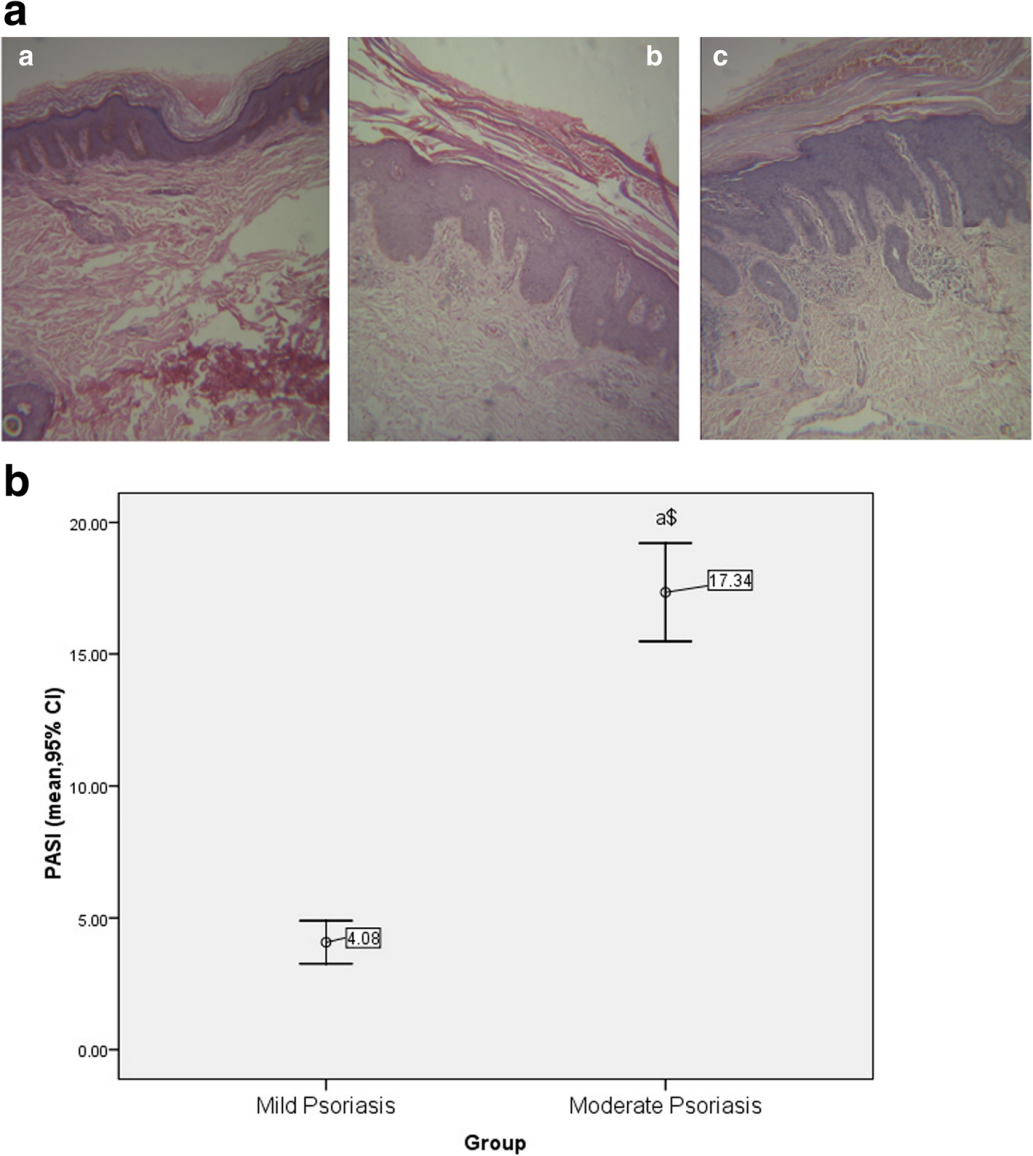
Histological and clinical examinations of mild and moderate psoriatic skin. **a** Histological examination of skin biopsy. (A) Photomicrograph of nonlesional skin biopsy. (B), (C) Photomicrograph of mild and moderate psoriasis vulgaris showing Munro microabscess, acanthosis, rete ridge elongation, and acute infiltration of dermis. **b** Clinical evidence of mild and moderate psoriatic lesion by PASI score. All values were expressed as mean ± SD. “a” denotes comparison of PASI score of mild and moderate psoriasis. The dollar sign denotes *P* < 0.001

The histological examination of nonlesional skin tissue shows normal rete ridges with stratum corneum whereas that of mild and moderate lesional skin revealed elongated rete ridges with deregulated stratum corneum (Fig. 1a). Histological analysis was further supported by PASI score. Forty-eight patients with mild psoriasis have a PASI score of 4.06 ± 2.46, and the remaining 48 patients with moderate psoriasis have a PASI score of 17.34 ± 5.64 (Fig. 1b).

### Mutated keratin sequences identified through Sanger sequencing

Almost in all the keratin mutation and sequence studies, NCBI database is widely used, NM_000526.4 (K14), NM_000421.3 (K10), NM_005557.3 (K16), and NM_000422.2 (K17), as publicly available sequence data for healthy population (control). According to the above keratins, nucleotide accession number of these sequences is also retrieved from the Chinese population [35–39]. So, in order to predict the mutation in two stages of psoriasis, we have taken the K14, K10, K16, and K17 coding sequences of healthy population available in NCBI database with accession numbers NM_000526.4 (K14), NM_000421.3 (K10), NM_005557.3 (K16), and NM_000422.2 (K17), which were further aligned respectively with the Sanger sequences of K14, K10, K16, and K17 obtained in our patients by using BioEdit software (Additional file 1: Figs. S1 and S2). In mild psoriasis patients, we have identified nine and seven missense mutations in K14 and K10, respectively, as well as no missense mutation was found in K16 and K17. However, in moderate psoriasis, we found nine, six, five, and eight mutations of K14, K10, K16, and K17, respectively.

Some of our mutated K14, K10, and K17 sequences were matched to previously reported sequences in other skin disease (Table 2). In order to compare the mutation identified in psoriasis patients with other skin disease patients, we have used the following Clinvar data in the NCBI website (https://www.ncbi.nlm.nih.gov/clinvar/) (Tables 3 and 4). According to the Clinvar data of NM_000526.4 (K14), 11 missense mutations of K14 were found in EBS disease; comparing these mutations with our K14 results, three mutations were found to be identical in both diseases. Likewise, NM_000421.3 (K10) compared with K10 of our patients showed only one identical mutation. Compared with our results, no identical mutation was found in both keratin 16 and 17. Based on per nucleotide sequence variation, percentage of background mutation rate was calculated. The high background mutation rates of K14 mutated nucleotide sequences in our patients were 33.3% G > C, followed by 22.2% A > T, 16.6% C > T, 11.1% G > T, and 5.5% of C > A, G > A, and C > G, whereas EBS disease condition obtained from the NCBI website showed high mutation rate of 36.3% G > A. Background mutation rates of psoriasis K10 were as follows: G > T, T > A, G > C, A > C, C > G, and C > A with percentages of 30.7, 15.3, and 7.6%. In EI disease, A > C nucleotide K10 mutation is found to be 23% followed with 15.3% of G > A and T > C. The most highest K16 mutated nucleotide found in our study is 40% of C > G whereas 25% of A > T, C > T, and G > T was found in K17.

**Table 2:** Mutated epidermal and hyperproliferative keratin sequences identified in this study consistent with previous reports (clinvar and dbSNP in the NCBI website)

S. no.	Gene	Mutation region	Accession number	
			Clinvar	dbSNP
1	Keratin 14	C442 > T	RCV000056740.1	rs58378809
2		A1234 > T	RCV000056680.1	rs267607403
3		G1237 > A	RCV000056682.1	rs59780231
4		C1246 > T		rs777067461
5	Keratin 10	C520 > A	RCV000056502.1	
6	Keratin 17	C986 > T		rs780535087

**Table 3:** Comparison of publicly available mutated keratin 14 and keratin 10 sequence data of various skin disease patients with mutated sequence obtained in our study (psoriasis) from Clinvar NCBI database

S. no.	Name	Gene(s)	Condition(s)	Clinical significance (Last reviewed)	GRCh37		GRCh38		Variation ID	Allele ID(s)
					Chromosome	Location	Chromosome	Location		
1	NM_000526.4(KRT14):c.1264G > A (p.Glu422Lys)	KRT14	EBH-DM	Pathogenic (Apr 1, 2000)	17	39739497	17	41583245	14623	29662
2	NM_000526.4(KRT14):c.1256T > A (p.Leu419Gln)	KRT14	EBH-DM	Pathogenic (Apr 1, 2000)	17	39739505	17	41583253	14622	29661
3	NM_000526.4(KRT14):c.1243T > C (p.Tyr415His)	KRT14	EBH-DM	Pathogenic (Mar 27, 2015)	17	39739518	17	41583266	14621	29660
*4*	*NM_000526.4(KRT14):c.1237G > A (p.Ala413Thr)*	*KRT 14*	*Not provided*	*Pathogenic (Jul 31, 2012)*	*17*	*39739524*	*17*	*41583272*	*66319*	*77216*
*5*	*NM_000526.4(KRT14):c.1234A > T (p.Ile412Phe)*	*KRT14*	*Not provided*	*Pathogenic (Apr 2, 2018)*	*17*	*39739527*	*17*	*41583275*	*66317*	*77214*
6	NM_000526.4(KRT14):c.1228C > T (p.Gln410Ter)	KRT14	Not provided	Pathogenic (Sep 15, 2016)	17	39739533	17	41583281	66313	77210
7	NM_000526.4(KRT14):c.1162C > T (p.Arg388Cys)	KRT14	Not provided	Pathogenic (Oct 13, 2017)	17	39739599	17	41583347	66306	77203
8	NM_000526.4(KRT14):c.1151T > C (p.Leu384Pro)	KRT14	EBS, Koebner type	Pathogenic (Nov 22, 1991)	17	39739610	17	41583358	14611	29650
*9*	*NM_000526.4(KRT14):c.442C > T (p.Arg148Cys)*	*KRT14*	*EBS, Koebner type*	*Pathogenic (Apr 2, 2018)*	*17*	*39742645*	*17*	*41586393*	*66368*	*77265*
10	NM_000526.4(KRT14):c.374G > A (p.Arg125His)	KRT14	EBH-DM	Pathogenic (Oct 30, 2017)	17	39742713	17	41586461	14613	29652
11	NM_000526.4(KRT14):c.357G > A (p.Met119Ile)	KRT14	EBS, autosomal recessive	Pathogenic (Sep 1, 1997)	17	39742730	17	41586478	14620	29659
12	NM_000421.3(KRT10):c.1374-2A > C	KRT10	Not provided	Pathogenic (Nov 25, 2015)	17	38975415	17	40819163	449615	445767
13	NM_000421.3(KRT10):c.1374-2A > G	KRT10	EI, congenital reticular	Pathogenic (Oct 1, 2010)	17	38975415	17	40819163	14581	29620
14	NM_000421.3(KRT10):c.1373 + 2T > C	KRT10	Not provided	Pathogenic (May 9, 2017)	17	38975767	17	40819515	432261	426219
15	NM_000421.3(KRT10):c.1373 + 1G > A	KRT10	EI, congenital reticular	Pathogenic (Oct 1, 2010)	17	38975768	17	40819516	14582	29621
16	NM_000421.3(KRT10):c.1325T > A (p.Leu442Gln)	KRT10	BIE	Pathogenic (Feb 1, 1994)	17	38975817	17	40819565	14575	29614
17	NM_000421.3(KRT10):c.1300C > T (p.Gln434Ter)	KRT10	BIE	Pathogenic (Apr 1, 2006)	17	38975842	17	40819590	29764	38719
18	NM_000421.3(KRT10):c.1281C > A (p.Cys427Ter)	KRT10	BIE	Pathogenic (Jul 1, 2008)	17	38975861	17	40819609	29765	38720
19	NM_000421.3(KRT10):c.494G > C (p.Arg165Pro)	KRT10	Not provided	Likely pathogenic (Sep 1, 2016)	17	38978344	17	40822092	432167	426220
20	NM_000421.3(KRT10):c.482T > C (p.Leu161Ser)	KRT10	BIE	Pathogenic (Aug 21, 1992)	17	38978356	17	40822104	14569	29608
*21*	*NM_000421.3(KRT10):c.479A > C (p.Tyr160Ser)*	*KRT10*	*Not provided*	*Pathogenic (Apr 2, 2018)*	*17*	*38978359*	*17*	*40822107*	*66178*	*77075*
22	NM_000421.3(KRT10):c.478T > G (p.Tyr160Asp)	KRT10	BIE	Pathogenic (Feb 1, 1994)	17	38978360	17	40822108	14572	29611
23	NM_000421.3(KRT10):c.467G > A (p.Arg156His)	KRT10	BIE	Pathogenic (Sep 20, 2017)	17	38978371	17	40822119	14573	29612
24	NM_000421.3(KRT10):c.460A > C (p.Asn154His)	KRT10	BIE	Pathogenic (Feb 1, 1994)	17	38978378	17	40822126	14571	29610

*EBH-DM* epidermolysis bullosa herpetiformis-Dowling Meara, *EBS* epidermolysis bullosa simplex, *EI* erythroderma ichthyosiform, *BIE* Bullous ichthyosiform erythroderma

Italicized words indicate comparison of identical mutation obtained in our study (psoriasis) with other skin disease

**Table 4:** Comparison of publicly available mutated keratin 16 and keratin 17 sequence data of various skin disease patients with mutated sequence obtained in our study (psoriasis) from Clinvar NCBI database

S. no.	Name	Gene(s)	Condition(s)	Clinical significance (last reviewed)	GRCh37		GRCh38		Variation ID	Allele ID(s)
					Chromosome	Location	Chromosome	Location		
1	NM_005557.3(KRT16):c.395T > C (p.Leu132Pro)	KRT16	PC 1	Pathogenic (Jan 29, 2016)	17	39768546	17	41612294	14600	29639
2	NM_005557.3(KRT16):c.379C > G (p.Arg127Gly)	KRT16	Not provided	Pathogenic (May 11, 2015)	17	39768562	17	41612310	265217	260165
3	NM_000422.2(KRT17):c.1163T > C (p.Leu388Pro)	KRT17	Not provided	Not provided	17	39776929	17	41620677	66181	77078
4	NM_000422.2(KRT17):c.1112T > C (p.Leu371Pro)	KRT17	Not provided	Not provided	17	39776980	17	41620728	66180	77077
5	NM_000422.2(KRT17):c.325A > G (p.Asn109Asp)	KRT17	Not provided	Not provided	17	39780437	17	41624185	66188	77085
6	NM_000422.2(KRT17):c.309T > C (p.Arg103=)	KRT17	Not provided	Not provided	17	39780453	17	41624201	66187	77084
7	NM_000422.2(KRT17):c.304G > A (p.Val102Met)	KRT17	PC 2	Pathogenic (Mar 1, 2002)	17	39780458	17	41624206	14599	29638
8	NM_000422.2(KRT17):c.296T > C (p.Leu99Pro)	KRT17	PC 2	Pathogenic (Jun 9, 2015)	17	39780466	17	41624214	14598	29637
9	NM_000422.2(KRT17):c.292T > G (p.Tyr98Asp)	KRT17	PC 2	Pathogenic (Feb 1, 1997)	17	39780470	17	41624218	14588	29627
10	NM_000422.2(KRT17):c.284T > C (p.Leu95Pro)	KRT17	PC 2	Pathogenic (May 14, 2015)	17	39780478	17	41624226	14596	29635
11	NM_000422.2(KRT17):c.284T > A (p.Leu95Gln)	KRT17	PC 2	Pathogenic (May 1, 2001)	17	39780478	17	41624226	14595	29634
12	NM_000422.2(KRT17):c.281G > C (p.Arg94Pro)	KRT17	PC 2	Pathogenic (May 1, 2001)	17	39780481	17	41624229	14594	29633
13	NM_000422.2(KRT17):c.281G > A (p.Arg94His)	KRT17	PC 2| SM	Pathogenic (Dec 1, 2001)	17	39780481	17	41624229	14590	29629
14	NM_000422.2(KRT17):c.280C > T (p.Arg94Cys)	KRT17	PC 2|	Pathogenic(Aug 15, 2016)	17	39780482	17	41624230	14591	29630
15	NM_000422.2(KRT17):c.275A > G (p.Asn92Ser)	KRT17	PC 2	Pathogenic (Jul 26, 2016)	17	39780487	17	41624235	14587	29626
16	NM_000422.2(KRT17):c.274A > C (p.Asn92His)	KRT17	SM	Pathogenic (Feb 1, 1997)	17	39780488	17	41624236	14589	29628
17	NM_000422.2(KRT17):c.274A > G (p.Asn92Asp)	KRT17	PC 2	Pathogenic (Nov 3, 2016)	17	39780488	17	41624236	14586	29625
18	NM_000422.2(KRT17):c.263T > A (p.Met88Lys)	KRT17	Not provided	Not provided	17	39780499	17	41624247	66182	77079
19	NM_000422.2(KRT17):c.263T > C (p.Met88Thr)	KRT17	PC 2	Pathogenic (Nov 1, 1999)	17	39780499	17	41624247	14592	29631
20	NM_000422.2(KRT17):c.-6G > C	KRT17	Not specified	Benign (Apr 28, 2016)	17	39780767	17	41624515	380281	375886

*PC* pachyonychia congenita, *SM* steatocystoma multiplex

### Validation of mutation and their pathogenicity using Mutation Surveyor and Alamut Visual software

**Fig. 2:**
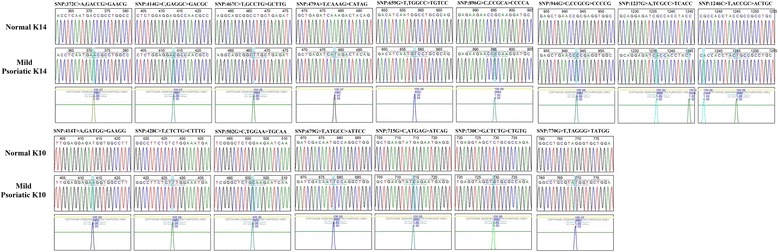
Sanger sequencing of mutated K14 and K10 in mild psoriasis analyzed by Mutation Surveyor. Sanger sequencing of K14 and K10 CDS region of mild psoriatic samples in scf format and reference sequence of NCBI CDS region of all keratins in Gb file format were analyzed by using Mutation Surveyor; the positions are annotated according to the genetic code

**Fig. 3:**
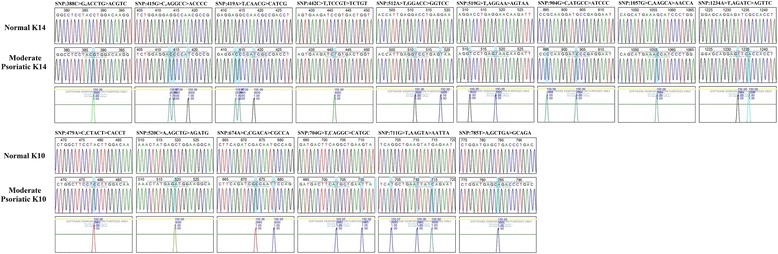
Sanger sequencing of mutated K14 and K10 in moderate psoriasis analyzed by Mutation Surveyor**.** Sanger sequencing of K14 and K10 CDS region of moderate psoriatic samples in scf format and reference sequence of NCBI CDS region of all keratins in Gb file format were analyzed by using Mutation Surveyor; the positions are annotated according to the genetic code

**Fig. 4:**
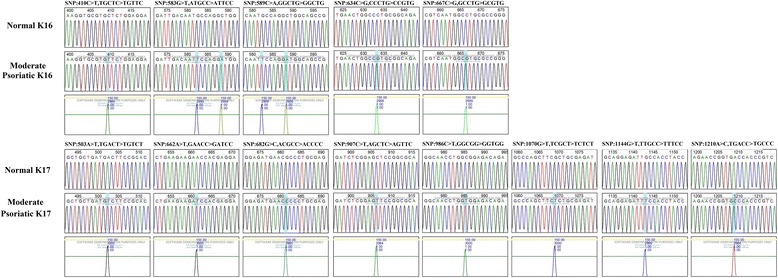
Sanger sequencing of mutated K16 and K17 in moderate psoriasis analyzed by Mutation Surveyor. Sanger sequencing of K16 and K17 CDS region of moderate psoriatic samples in scf format and reference sequence of NCBI CDS region of all keratins in Gb file format were analyzed by using Mutation Surveyor; the positions are annotated according to the genetic code

To validate and confirm the mutation, we did in silico analysis of all keratin sequences obtained in these patients by using Mutation Surveyor software. Similar to Sanger sequence results, in silico analysis also showed nine and seven missense mutations in K14 and K10, respectively, as well as no missense mutation was found in K16 and K17 in mild psoriasis (Fig. 2). Also, in moderate psoriasis, a similar mutation pattern was shown (Figs. 3 and 4) using Mutation Surveyor software. The mutation’s pathogenicity was predicted by using Alamut Visual software; we found about 34 and 15% of K14 and K10 mutated sequences in mild psoriasis that were predicted to be tolerated, and the remaining sequences were predicted to have damaging effect. Based on Mutation Assessor, all mutated sequences in mild psoriasis have medium effect on the alteration of these protein functions (Table 5; Additional file 1: Fig. S1) where, as in moderate psoriasis, all mutated sequences are predicted to have a damaging role with high effect on the alteration of protein functions (Table 6; Additional file 1: Fig. S2). Totally, we identified 44 mutations among which 16 mutations were found in both mild and moderate psoriasis, whereas 28 mutations were found only in moderate psoriasis.

**Table 5:** Prediction of epidermal keratin mutation’s pathogenicity in mild psoriasis using Alamut Visual software

S. no.	MT position	Exon	seq.MT	Type of MT	AA change	Variant in protein domain	SIFT			Mutation taster			Mutation assessor		
							Prediction	Score	Range score	Prediction	Range score	*P* value	Prediction	Score	Range score
	K14														
1	372	1	C > A	Missense	p.(Asp124Glu)	IF	Damaging	0.017	0.5119	DC	0.4153	0.992	Medium	2.875	0.8364
2	414	1	G > C	Missense	p.(Glu138Asp)	IF	Tolerated	0.057	0.3792	DC	0.4441	0.998	Medium	2.0099	0.5492
3	467	1	C > T	Missense	p.(Pro156Leu)	IF	Damaging	0.006	0.6138	DC	0.8103	1	Medium	3.335	0.9109
4	479	1	A > T	Missense	p.(Lys160Ile)	IF	Damaging	0.019	0.5008	Polymorphism	0.08979	1	Medium	3.125	0.8823
5	659	2	G > T	Missense	p.(Gly220Val)	IF protein, keratin type I	Damaging	0	0.9122	DC	0.8103	1	Medium	2.565	0.7524
6	896	4	G > C	Missense	p.(Arg299Pro)	IF protein, prefoldin	Damaging	0.008	0.5857	DC	0.4637	0.999	Medium	3.1099	0.8799
7	944	4	G > C	Missense	p.(Arg315Pro)	IF protein, prefoldin	Damaging	0.006	0.6138	DC	0.3752	0.946	Medium	2.3399	0.6737
8	1237	6	G > A	Missense	p.(Ala413Thr)	IF protein (conserved site), IF	Tolerated	0.099	0.3061	Polymorphism	0.4614	0.001	Medium	2.2999	0.6591
9	1246	6	C > T	Missense	p.(Arg416Cys)	IF protein (conserved site), IF	Tolerated	0.132	0.2648	DC	0.8103	1	Medium	2.9349	0.8489
	K10														
10	414	1	T > A	Missense	p.(Asp138Glu)		Tolerated	0.828	0.02894	DC, polymorphism	0.8103	0.992	Medium	2.1549	0.6042
11	428	1	C > T	Missense	p.(Ser143Phe)		Damaging	0.016	0.518	DC	0.8103	0.55	Medium	2.14	0.6007
12	502	1	G > C	Missense	p.(Glu168Gln)	IF protein	Damaging	0.005	0.6317	DC	0.8103	1	Medium	3.4849	0.9277
13	679	2	G > T	Missense	p.(Ala227Ser)	IF protein, keratin type I	Damaging	0.023	0.4813	DC	0.8103	1	Medium	2.6349	0.7736
14	715	2	G > C	Missense	p.(Glu239Gln)	IF protein, keratin type I	Damaging	0.001	0.7842	DC	0.8103	1	Medium	2.7799	0.8138
15	730	2	C > G	Missense	p.(Leu244Val)	IF protein	Damaging	0.005	0.6317	DC	0.8103	0.97	Medium	2.8199	0.8237
16	770	3	G > T	Missense	p.(Arg257Met)	IF protein, keratin type I	Damaging	0	0.9122	DC	0.8103	0.916	Medium	3.25	0.9002

*DC* disease causing, *IF* intermediate filament, *MT* mutation, *AA* amino acid

**Table 6:** Prediction of epidermal and hyperproliferative keratin mutation’s pathogenicity in moderate psoriasis using Alamut Visual software

S. no.	MT position	Exon	seq.MT	Type of MT	AA change	Variant in protein domain	SIFT			Mutation taster			Mutation assessor		
							Prediction	Score	Range score	Prediction	Range score	*P* value	Prediction	Score	Range score
	K14														
1	388	1	C > G	Missense	p.(Leu130Val)	IF protein	Damaging	0.001	0.7842	DC	0.537	1	High	3.8199	0.9566
2	415	1	G > C	Missense	p.(Ala139Pro)	IF protein	Damaging	0.006	0.6138	DC	0.8103	1	High	4.3049	0.9832
3	419	1	A > T	Missense	p.(Asn140Ile)	IF protein	Damaging	0	0.9122	DC	0.5881	1	High	4.425	0.9876
4	442	1	C > T	Missense	p.(Arg148Cys)	IF protein	Damaging	0	0.9122	DC	0.5881	1	High	3.75	0.9513
5	512	1	A > T	Missense	p.(Asp171Val)	IF protein	Damaging	0	0.9122	DC	0.8103	1	High	3.835	0.9576
6	519	1	G > T	Missense	p.(Arg173Ser)	IF protein	Damaging	0.001	0.7842	DC	0.4122	0.991	High	4.025	0.9697
7	904	4	G > C	Missense	p.(Ala302Pro)	IF protein, prefoldin	Damaging	0.001	0.7842	Polymorphism	0.2979	0.735	High	4.085	0.9729
8	1057	6	G > C	Missense	p.(Ala353Pro)	IF protein, keratin type I	Tolerated	0.189	0.2119	DC	0.4816	0.999	High	3.875	0.9604
9	1234	6	A > T	Missense	p.(Ile412Phe)	IF protein (conserved site), IF	Damaging	0	0.9122	DC	0.8103	1	High	4.65	0.9939
	K10														
10	479	1	A > C	Missense	p.(Tyr160Ser)	IF protein	Damaging	0	0.9122	DC	0.8103	1	High	4.5549	0.9916
11	520	1	C > A	Missense	p.(Leu174Met)	IF protein	Damaging	0.001	0.7842	DC	0.8103	0.991	High	3.5099	0.9302
12	674	2	A > C	Missense	p.(Asp225Ala)	IF protein	Damaging	0	0.9122	DC	0.8103	1	High	3.9549	0.9656
13	704	2	G > T	Missense	p.(Arg235Met)	IF protein, keratin type I	Damaging	0	0.9122	DC	0.8103	0.84	High	4.2849	0.9824
14	711	2	G > T	Missense	p.(Lys237Asn)	IF protein, keratin type I	Damaging	0	0.9122	DC	0.8103	0.983	High	3.66	0.944
15	785	3	T > A	Missense	p.(Leu262Gln)	IF protein, prefoldin, keratin I	Damaging	0	0.9122	DC	0.8103	0.834	High	4.2399	0.9805
	K16														
16	410	1	C > T	Missense	p.(Ala137Val)	IF	Damaging	0.002	0.7209	Polymorphism	0.3052	0.65	Medium	3.39	0.9174
17	583	1	G > T	Missense	p.(Ala195Ser)	IF protein, keratin type I	Damaging	0.029	0.457	DC	0.4734	1	Medium	2.805	0.8202
18	589	1	C > A	Missense	p.(Leu197Met)	IF protein, keratin type I	Damaging	0.028	0.4608	DC	0.4467	0.998	Medium	2.88	0.8371
19	634	1	C > G	Missense	p.(Leu212Val)	IF	Damaging	0.009	0.5743	DC	0.4067	0.988	Medium	2.9849	0.8585
20	667	1	C > G	Missense	p.(Leu223Val)	IF protein, keratin type I	Damaging	0.001	0.7842	DC	0.4434	1	Medium	3.3	0.9406
	K17														
21	503	1	A > T	Missense	p.(Asp168Val)	IF protein, keratin type I	Damaging	0	0.9122	DC	0.8103	1	Medium	3.3	0.9473
22	662	2	A > T	Missense	p.(Asn221Ile)	IF protein, prefoldin	Damaging	0.01	0	Polymorphism	0.4721	0.999	Medium	2.605	0.7645
23	682	3	G > C	Missense	p.(Ala228Pro)	IF protein, prefoldin	Damaging	0.016	0.518	Polymorphism	0.1948	1	Medium	3.25	0.9409
24	907	4	C > T	Missense	p.(Leu303Phe)	IF protein, prefoldin	Damaging	0.03	0, 0.001	Polymorphism	0.5164	1	Medium	3.4749	0.9267
26	986	5	C > T	Missense	p.(Ala329Val)	IF protein	Damaging	0.51	0.017	Polymorphism	0.4593	0.999	Medium	3.17	0.8891
26	1070	5	G > T	Missense	p.(Arg357Leu)	IF protein, keratin type I	Damaging	0	0.9122	DC	0.8103	1	Medium	3.17	0.9796
27	1144	6	G > T	Missense	p.(Ala382Ser)	IF protein (conserved site), IF	Tolerated	0	0.122	Polymorphism	0.4437	0.998	Medium	2.265	0.6465
28	1210	6	A > C	Missense	p.(Thr404Pro)	IF protein (conserved site), IF	Tolerated	0	0.075	Polymorphism	0.5199	1	Medium	2.6099	0.7658

*DC* disease causing, *IF* intermediate filament, *MT* mutation, *AA* amino acid

### Comparison of mutated keratins with ExAC databases

When we compared K14, K10, K16, and K17 missense mutated variants in ExAC database with their respective K14, K10, K16, and K17 mutated variants observed in our psoriasis patients, we perceived that one K14 c1237G > A variant with MAF 0.01 and one K17 c986C > T variant with MAF < 0.001 existed in the ExAC database; additionally, none of the K10 and K16 variants were found to occur in the ExAC database. Based on the analysis, our result showed that 99% of K14 and K17 and 100% of K10 and K16 missense mutations in our psoriasis patients were considered to be as novel missense mutation which did not exist in the ExAC database. These novel mutations might be one of the reasons causing severe damage in psoriasis patients (Additional file 2: Table S1).

### Identification of deleterious mutations

Keratin 14, 10, 16, and 17 CDS region of amino acid sequence in Fasta format and 44 missense mutations, which were obtained from Mutation Surveyor and Visual Alamut, were loaded to PredictSNP1.0, and all available integrated tools were selected for prediction. PredictSNP1.0 provided predictions for each integrated tool and a consensus prediction as percentages (expected accuracies) and the effect of mutation on protein function as “neutral” and “deleterious” by PredictSNP and all the integrated tools, except for nsSNPAnalyzer, which did not give any prediction for any mutations. In mild psoriasis, about 70% of mutated sequences in both K14 and K10 were found to be deleterious, whereas in moderate psoriasis, all K14 and K10; one K16 and six K17 mutated sequences were found to be deleterious. Out of 44 mutations, 33 mutations were predicted to be as deleterious by PredictSNP and all the integrated tools, except for nsSNPAnalyzer, which did not give any prediction for any mutations (Tables 7 and 8).

**Table 7:** The expected accuracy results of the missense mutations of epidermal keratins in mild psoriasis predicted as deleterious in PredictSNP and integrated tool

Gene	CDS mutation	AA mutation	Predict SNP		MAPP		PhD-SNP		PolyPhen-1		PolyPhen-2		SIFT		SNAP		PANTHER	
			Prediction	Accuracy	Prediction	Accuracy	Prediction	Accuracy	Prediction	Accuracy	Prediction	Accuracy	Prediction	Accuracy	Prediction	Accuracy	Prediction	Accuracy
K14	c.372C > A	p.D124E	DEL	0.55	DEL	0.59	DEL	0.68	NEU	0.67	DEL	0.59	DEL	0.53	NEU	0.67	DEL	0.69
	c.414G > C	p.E138D	NEU	0.65	NEU	0.64	DEL	0.59	NEU	0.67	NEU	0.72	DEL	0.43	NEU	0.71	DEL	0.66
	c.467C > T	p.P156L	NEU	0.60	DEL	0.48	NEU	0.66	NEU	0.67	DEL	0.40	DEL	0.53	NEU	0.58	DEL	0.67
	c.479A > T	p.K160I	DEL	0.55	DEL	0.41	NEU	0.66	DEL	0.74	NEU	0.61	DEL	0.45	DEL	0.62	DEL	0.57
	c.659G > T	p.G220V	DEL	0.51	NEU	0.73	NEU	0.45	DEL	0.74	DEL	0.65	DEL	0.79	NEU	0.55	DEL	0.74
	c.896G > C	p.R299P	DEL	0.87	DEL	0.75	DEL	0.88	DEL	0.59	DEL	0.50	DEL	0.53	DEL	0.72	DEL	0.74
	c.944G > C	p.R315P	DEL	0.76	DEL	0.62	DEL	0.82	DEL	0.59	DEL	0.50	DEL	0.79	NEU	0.50	DEL	0.70
	c.1237G > A	p.A413T	NEU	0.65	DEL	0.62	NEU	0.51	NEU	0.67	DEL	0.40	NEU	0.67	NEU	0.71	NEU	0.47
	c.1246C > T	p.R416C	DEL	0.61	DEL	0.57	DEL	0.88	DEL	0.59	DEL	0.50	NEU	0.71	NEU	0.55	DEL	0.78
K10	c.414T > A	p.D138E	NEU	0.83	NEU	0.77	NEU	0.66	0	0	0	0	NEU	0.81	NEU	0.83	NEU	0.67
	c.428C > T	p.S143F	NEU	0.60	NEU	0.75	DEL	0.61	0	0	0	0	DEL	0.53	NEU	0.55	DEL	0.74
	c.502G > C	p.E168Q	DEL	0.76	DEL	0.59	DEL	0.86	DEL	0.59	DEL	0.63	DEL	0.53	NEU	0.50	DEL	0.74
	c.679G > T	p.A227S	DEL	0.52	DEL	0.41	DEL	0.77	NEU	0.67	DEL	0.50	DEL	0.46	NEU	0.71	DEL	0.57
	c.715G > C	p.E239Q	DEL	0.76	DEL	0.48	DEL	0.68	DEL	0.59	DEL	0.63	DEL	0.79	NEU	0.55	DEL	0.74
	c.730C > G	p.L244V	DEL	0.55	DEL	0.59	DEL	0.68	NEU	0.67	DEL	0.50	DEL	0.53	NEU	0.71	DEL	0.66
	c.770G > T	p.R257M	DEL	0.76	DEL	0.51	DEL	0.73	DEL	0.74	DEL	0.54	DEL	0.79	NEU	0.58	DEL	0.74

*DEL* deleterious, *NEU* neutral

**Table 8:** The expected accuracy results of the missense mutations of epidermal and hyperproliferative keratins in moderate psoriasis predicted as deleterious in PredictSNP and integrated tool

Gene	CDS mutation	AA mutation	Predict SNP		MAPP		PhD-SNP		PolyPhen-1		PolyPhen-2		SIFT		SNAP		PANTHER	
			Prediction	Accuracy	Prediction	Accuracy	Prediction	Accuracy	Prediction	Accuracy	Prediction	Accuracy	Prediction	Accuracy	Prediction	Accuracy	Prediction	Accuracy
K14	c.388C > G	p.L130V	DEL	0.76	DEL	0.72	DEL	0.86	DEL	0.74	DEL	0.65	DEL	0.79	NEU	0.58	DEL	0.77
	c.415G > C	p.A139P	DEL	0.87	DEL	0.59	DEL	0.82	DEL	0.74	DEL	0.65	DEL	0.53	DEL	0.56	DEL	0.74
	c.419A > T	p.N140I	DEL	0.87	DEL	0.84	DEL	0.86	DEL	0.74	DEL	0.81	DEL	0.79	DEL	0.62	DEL	0.87
	c.442C > T	p.R148C	DEL	0.76	DEL	0.59	DEL	0.88	DEL	0.74	DEL	0.68	DEL	0.79	NEU	0.55	DEL	0.87
	c.512A > T	p.D171V	DEL	0.76	DEL	0.59	DEL	0.82	DEL	0.74	DEL	0.56	DEL	0.79	NEU	0.50	DEL	0.74
	c.519G > T	p.R173S	DEL	0.76	DEL	0.59	DEL	0.61	DEL	0.59	DEL	0.43	DEL	0.79	NEU	0.50	DEL	0.74
	c.904G > C	p.A302P	DEL	0.61	DEL	0.75	DEL	0.82	DEL	0.59	NEU	0.64	DEL	0.79	NEU	0.50	DEL	0.84
	c.1057G > C	p.A353P	DEL	0.61	DEL	0.57	DEL	0.86	DEL	0.74	DEL	0.47	NEU	0.74	NEU	0.61	DEL	0.68
	c.1234A > T	P.I412F	DEL	0.87	DEL	0.63	DEL	0.77	DEL	0.74	DEL	0.81	DEL	0.79	DEL	0.56	–	0.00
K10	c.479A > C	p.Y160S	DEL	0.87	DEL	0.86	DEL	0.86	DEL	0.74	DEL	0.81	DEL	0.79	DEL	0.72	–	0.00
	c.520C > A	p.L174M	DEL	0.76	DEL	0.72	DEL	0.59	DEL	0.74	DEL	0.65	DEL	0.79	NEU	0.55	DEL	0.72
	c.674A > C	p.D225A	DEL	0.87	DEL	0.66	DEL	0.68	DEL	0.74	DEL	0.68	DEL	0.79	DEL	0.56	DEL	0.77
	c.704G > T	p.R235M	DEL	0.76	DEL	0.59	DEL	0.82	DEL	0.74	DEL	0.81	DEL	0.79	NEU	0.55	DEL	0.78
	c.711G > T	p.K237N	DEL	0.76	DEL	0.75	DEL	0.88	DEL	0.59	DEL	0.63	DEL	0.79	NEU	0.50	DEL	0.74
	c.785T > A	p.L262Q	DEL	0.76	DEL	0.41	DEL	0.61	DEL	0.74	DEL	0.81	DEL	0.79	NEU	0.50	DEL	0.78
K16	c.410C > T	p.A137V	NEU	0.63	NEU	0.70	NEU	0.45	DEL	0.59	NEU	0.61	DEL	0.79	NEU	0.71	DEL	0.68
	c.583G > T	p.A195S	NEU	0.65	NEU	0.66	NEU	0.51	NEU	0.67	DEL	0.47	DEL	0.45	NEU	0.71	DEL	0.72
	c.589C > A	p.L197M	NEU	0.60	NEU	0.79	DEL	0.59	NEU	0.67	DEL	0.59	DEL	0.45	NEU	0.83	DEL	0.67
	c.634C > G	p.L212V	NEU	0.60	DEL	0.51	NEU	0.51	NEU	0.67	DEL	0.45	DEL	0.53	NEU	0.77	DEL	0.71
	c.667C > G	p.L223V	DEL	0.76	DEL	0.75	DEL	0.77	DEL	0.74	DEL	0.65	DEL	0.79	NEU	0.55	DEL	0.71
K17	c.503A > T	p.D168V	DEL	0.87	DEL	0.63	DEL	0.82	DEL	0.59	DEL	0.68	DEL	0.79	DEL	0.56	DEL	0.87
	c.662A > T	p.N221I	DEL	0.72	NEU	0.77	DEL	0.59	DEL	0.74	DEL	0.81	DEL	0.79	DEL	0.56	DEL	0.74
	c.682G > C	p.A228P	DEL	0.72	DEL	0.48	DEL	0.88	DEL	0.74	DEL	0.50	DEL	0.46	NEU	0.61	DEL	0.73
	c.907C > T	p.L303F	DEL	0.51	NEU	0.75	NEU	0.51	DEL	0.74	DEL	0.65	DEL	0.79	NEU	0.50	DEL	0.78
	c.986C > T	p.A329V	DEL	0.55	DEL	0.48	DEL	0.77	NEU	0.67	DEL	0.40	DEL	0.46	NEU	0.61	DEL	0.72
	c.1070G > T	p.R357L	DEL	0.72	DEL	0.77	DEL	0.68	DEL	0.59	NEU	0.68	DEL	0.79	DEL	0.56	DEL	0.76
	c.1144G > T	p.A382S	NEU	0.83	NEU	0.64	NEU	0.51	NEU	0.67	NEU	0.70	NEU	0.71	NEU	0.71	DEL	0.57
	c.1210A > C	p.T404P	NEU	0.63	DEL	0.51	DEL	0.68	NEU	0.67	NEU	0.71	NEU	0.65	NEU	0.50	NEU	0.48

*DEL* deleterious, *NEU* neutral

### Location of the deleterious mutations on the secondary structure and 3D structure of keratin protein

**Fig. 5:**
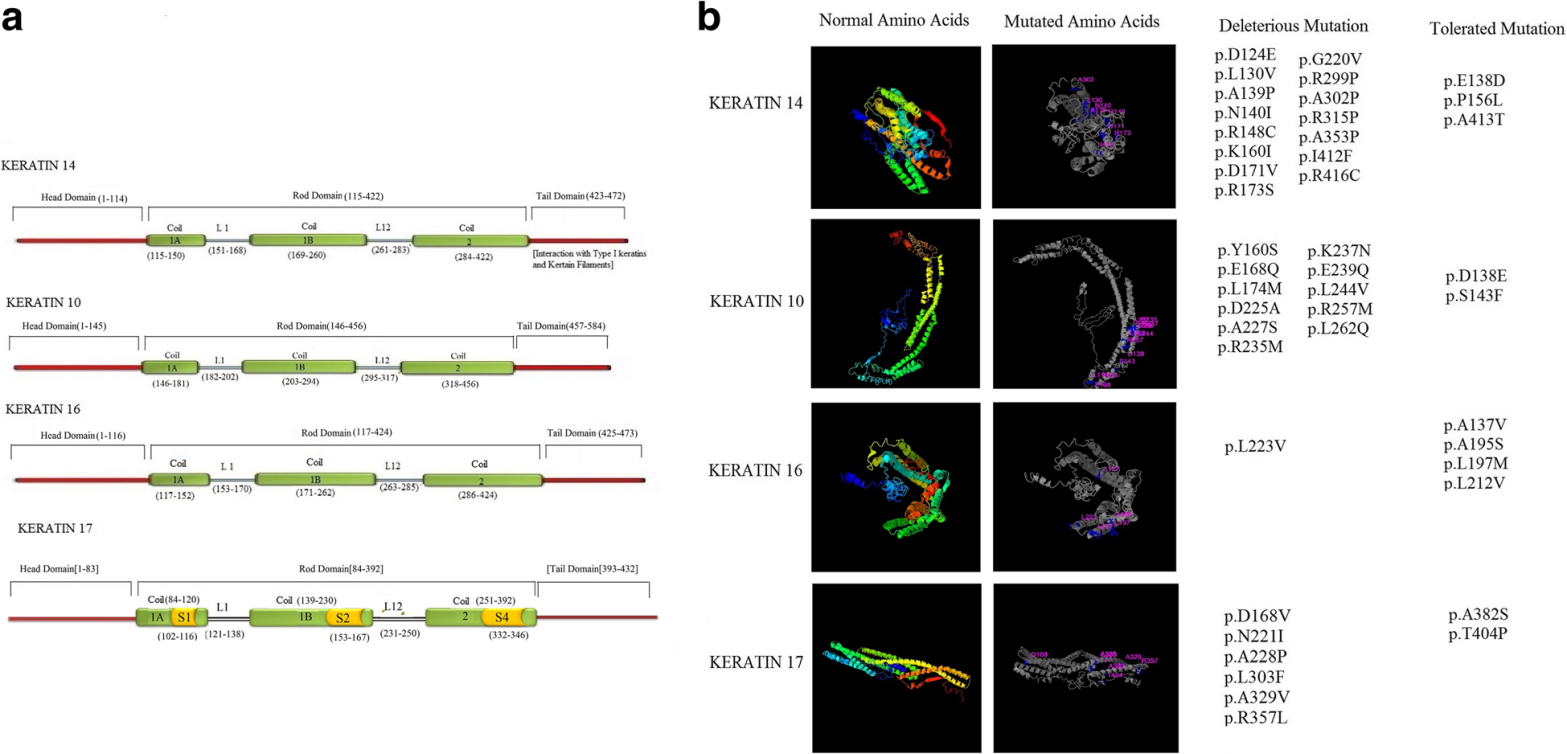
Protein structure of epidermal and hyperproliferative keratins. **a** Schematic representation of secondary structure of all four keratins with their domain and sub-domain, whereas, S = peptide epitope and L = linker. **b** Specific regions of 33 deleterious mutation localizations. The figures in the left side are the 3D structure of all four keratin CDS regions. The figures in the right side are the 3D structures of domains with mutated residues. The mutated residues are numbered according to their position on the regions. The position of mutated amino acids (aa) on all four keratins and on CDS regions are provided

Position of these deleterious mutations in the keratin secondary and 3D structure is important because amino acid sequences of keratin are mainly involved in the assembly of keratin filaments and also binding of keratins and keratin filaments to cell adhesion complexes or signaling molecules. The secondary structure of these keratin proteins has three sub-domains, namely, head domain, central α-helical rod domain, and tail domain. The rod domain is mainly composed of sub-domain coils 1A, 1B, 2A, and 2B connected by linkers L1, L12, and L2 (Fig. 5a). We detected that these 33 mutations were located in the rod domain of these keratin proteins. The deleterious mutations in the rod domain were mainly noticed in coil 1 and 2 regions, which are critical for the protein activity and structure of these keratins.

Pathogenic K14 mutated sequences are mainly located in exon 1 and exon 6. In keratin 14, exon 1 (1–586 nucleotide) mainly forms the head and rod (coil 1A, L1, and 1B) region and exon 6 (1115–1335 nucleotide) forms the coil 2 of the rod domain. In K14, coil 1A region has five mutated amino acids, L1 region has one mutated amino acid, coil 1B has three mutated amino acids, and coil 2 region has six mutated amino acids. All appeared in close proximity in the folded protein (Fig. 5b).

Exons 1 and 2 are the major regions affected by the mutation in K10; they mainly form the head and rod domain region of 1A and 1B. We found three mutations in coil 1A region and eight mutations in coil 1B region of the rod domain. In K16, one mutation in coil 1B region was found to be deleterious. However, in K17, one mutation in coil 1B region of peptide epitope S2, two mutations in 1B region of peptide epitope S3, and three mutations in coil 2 region of peptide epitope S4 of the rod domain were found (Fig. 5b) The deleterious mutations in the rod domain of all these keratins were mainly noticed in coil 1 and 2 regions, which are critical for the protein activity and structure of these keratins.

Our results showed that the predicted deleterious mutations highly located in the α-helical rod domain, which forms a coiled structure to these keratins, are important to maintain the structural integrity of the skin.

### Effect of deleterious mutation on keratin protein stability

After predicting the deleterious effect and position of these mutated sequences in protein, it is imperative to analyze the stability of this protein. Therefore, we have analyzed the protein stability of these 33 deleterious mutated sequences on MUpro server and I-Mutant 2.0. Our results showed that in mild psoriasis, about two third of K14 and K10 mutation decrease the protein stability. However, in moderate stage, 90% of all mutated K14, K10, K16, and K17 sequences showed decreased protein stability (Table 9).

**Table 9:** Protein stability change prediction results of MUpro and I-Mutant2.0 for the 33 deleterious missense mutations of epidermal and hyperproliferative keratins in mild and moderate psoriasis patients

Stages of psoriasis	Gene	CDS mutation	AA mutation	MUpro				I-Mutant 2.0	
				SVM	SVMCS	NN	NNCS	Stability	DDG
Mild	K14	c.372C > A	p.D124E	Increase	0.05	Increase	0.71	Decrease	− 0.52
		c.479A > T	p.K160I	Decrease	− 0.54	Decrease	− 0.89	Decrease	0.61
		c.659G > T	p.G220V	Increase	0.41	Increase	0.65	Decrease	− 0.46
		c.896G > C	p.R299P	Decrease	− 0.57	Decrease	− 0.75	Decrease	− 2.22
		c.944G > C	p.R315P	Decrease	− 0.53	Decrease	− 0.86	Decrease	− 1.26
		c.1246C > T	p.R416C	Decrease	− 0.16	Decrease	− 0.58	Decrease	− 0.3
	K10	c.502G > C	p.E168Q	Increase	0.39	Increase	0.70	Decrease	− 0.01
		c.679G > T	p.A227S	Decrease	− 1	Decrease	− 0.71	Decrease	− 0.19
		c.715G > C	p.E239Q	Decrease	− 0.12	Decrease	− 0.64	Decrease	− 0.28
		c.730C > G	p.L244V	Decrease	− 1	Decrease	− 0.94	Decrease	− 2.03
		c.770G > T	p.R257M	Increase	0.53	Increase	0.81	Decrease	− 0.47
Moderate	K14	c.388C > G	p.L130V	Decrease	− 1	Decrease	− 1	Decrease	− 1.67
		c.415G > C	p.A139P	Decrease	− 0.63	Decrease	− 0.88	Decrease	− 1.89
		c.419A > T	p.N140I	Decrease	− 0.23	Decrease	− 0.67	Increase	1.57
		c.442C > T	p.R148C	Decrease	− 1	Decrease	− 1	Decrease	− 0.28
		c.512A > T	p.D171V	Decrease	− 0.37	Decrease	− 1	Decrease	− 0.36
		c.519G > T	p.R173S	Decrease	− 1	Decrease	− 1	Decrease	− 2.37
		c.904G > C	p.A302P	Increase	0.63	Increase	0.73	Decrease	− 3.34
		c.1057G > C	p.A353P	Decrease	− 0.58	Decrease	− 0.65	Increase	− 0.65
		c.1234A > T	P.I412F	Decrease	− 1	Decrease	− 0.69	Decrease	− 0.95
	K10	c.479A > C	p.Y160S	Decrease	− 0.47	Decrease	− 0.64	Decrease	− 1.16
		c.520C > A	p.L174M	Decrease	− 1	Decrease	− 1	Decrease	0.12
		c.674A > C	p.D225A	Decrease	− 0.39	Decrease	− 0.78	Decrease	− 0.76
		c.704G > T	p.R235M	Decrease	− 0.45	Decrease	− 0.89	Decrease	− 0.59
		c.711G > T	p.K237N	Decrease	− 0.60	Decrease	− 1	Increase	0.26
		c.785T > A	p.L262Q	Decrease	− 1	Decrease	− 1	Decrease	− 1.82
	K16	c.667C > G	p.L223V	Decrease	− 1	Decrease	− 1	Decrease	− 1.39
	K17	c.503A > T	p.D168V	Decrease	− 0.30	Decrease	− 0.60	Decrease	− 0.73
		c.662A > T	p.N221I	Decrease	− 0.70	Increase	0.62	Increase	1.64
		c.682G > C	p.A228P	Decrease	− 0.04	Increase	0.61	Decrease	− 2.48
		c.907C > T	p.L303F	Decrease	− 1	Decrease	− 1	Decrease	0.95
		c.986C > T	p.A329V	Decrease	− 0.34	Decrease	− 0.88	Decrease	0.12
		c.1070G > T	p.R357L	Increase	0.15	Increase	0.68	Increase	0.66

*CDS* coding sequences, *AA* amino acid, *SVM* support vector machine, *SVMCS* SVM confidence score, *NN* neural network, *NNCS* neural network confidence score, *DDG* free energy change value

### Immunofluorescence analysis of K14, K10, K16, and K17 antibodies

**Fig. 6:**
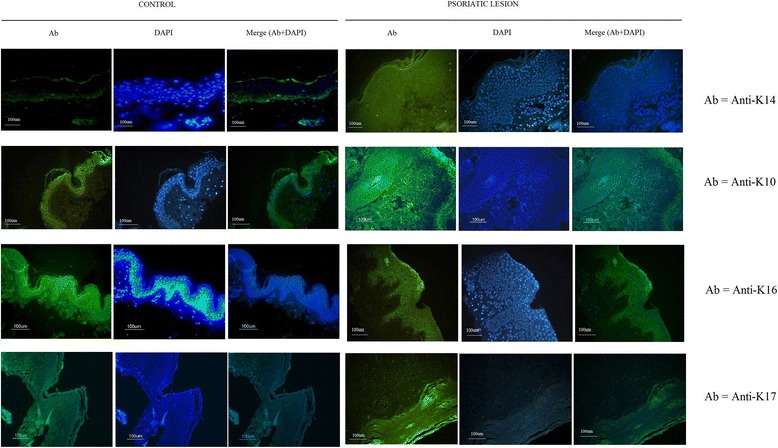
Immunofluorescence analysis of keratins in nonlesional and lesional psoriatic skin. Immunofluorescence analysis of epidermal and hyperproliferative type I keratins in frozen skin sections from patients with psoriasis in nonlesional and lesional skin (× 20 magnification, respectively), and nuclei were visualized with 4′-6-diamidino-2-phenylindole (DAPI). Bar = 100 μm; Ab antibody

Our immunofluorescence analysis of normal epidermis shows nuclei stained blue with DAPI throughout all the epidermal layers, whereas K14-positive cells are found only in the basal epidermal cell. Merging discloses co-expression of K14 and DAPI nuclear stain in most of the basal epidermal cells in control samples. In lesional samples, no staining of K14 was seen in the basal layer, whereas DAPI staining was seen throughout the elongated rete ridges, and merged results show there is no staining of K14 in the basal layer (Fig. 6).

DAPI stain was seen throughout all the epidermal layers, and K10 antibodies were stained in the spinosum of the epidermal layer in normal epidermis. K10-positive cells were seen marginally in the elongated rete ridges of the suprabasal epidermal layer. Merging of two stains shows marginal expression of K10 and strong DAPI staining in the nuclei of cells of the suprabasal epidermal layer (Fig. 6).

To confirm the expression of abnormal proteins K16 and K17 on the protein level, we performed immunofluorescence analyses in the corresponding control and lesional psoriatic skin samples. These analyses clearly reflected that there is no K16 expression, and blue DAPI stain stained the nuclei present in the normal epidermal layer. Psoriasis samples showed a strong expression of K16 throughout the suprabasal with nuclei stained with DAPI (Fig. 6). In addition, we analyzed there is no K17 protein in the normal epidermal layer, but nuclei were stained with DAPI and merging showed the blue nuclei staining throughout the epidermis, whereas in psoriasis, the K17 protein was seen in the stratum corneum along with DAPI staining (Fig. 6).

### Expression of keratins and their correlation with PASI in both mild and moderate psoriases

**Fig. 7:**
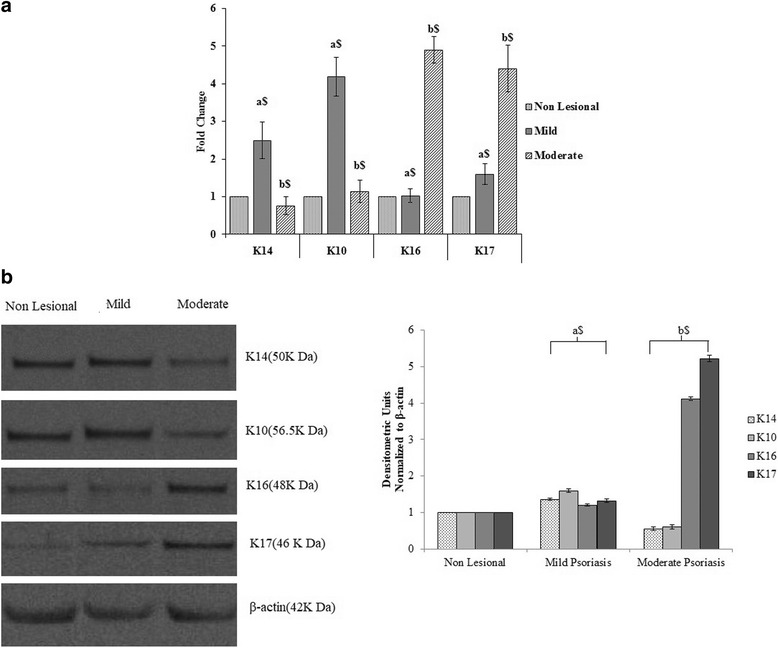
mRNA and protein expression of epidermal and hyperproliferative type I keratins in mild and moderate psoriasis. **a** Quantification of keratin 14, 10, 16, and 17 mRNA by real-time PCR using the comparative C_T_ method. The C_T_ values were normalized on the basis of GADPH and analyzed relative to that of the normal control. Gene expressions were given as fold change. **b** Western blot and densitometric analysis of keratins in mild and moderate psoriasis. All values were expressed as mean ± SD, where the dollar sign denotes *P* < 0.001, “a” denotes comparison of nonlesional with mild psoriatic lesional skin biopsy, and “b”: denotes comparison of mild and moderate psoriatic lesional skin biopsy

In mild psoriatic samples, the mRNA expression of K14 and K10 was significantly elevated to 2.5- and 4.4-folds compared to nonlesional, respectively, whereas in moderate case, these expressions were decreased significantly. In contrast to K14 and K10 expression, decrease in K16 and K17 expression was found in mild psoriasis; as the disease progresses in moderate psoriasis, the expression of K16 and K17 significantly elevated at 4.9- and 4.4-folds compared to nonlesional, respectively (Fig. 7a). Further, these results were confirmed by the Western blot analysis (Fig. 7b). We strongly believed that the changes in keratin expression in the psoriatic epidermis might be due to some pathogenic variants in these keratin genes.

**Fig. 8:**
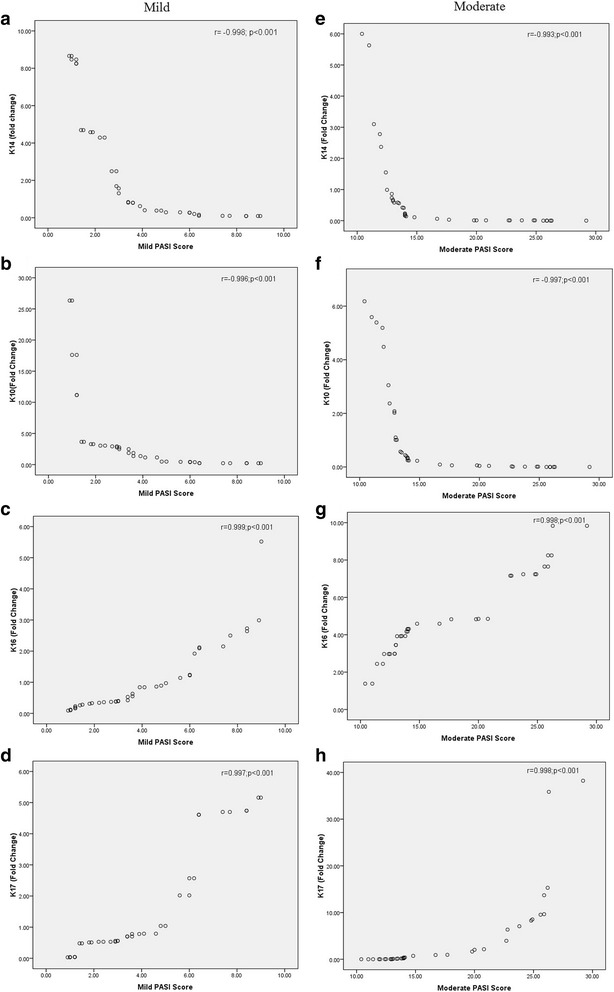
Correlation between keratins with PASI score. **a** Keratin 14 vs. mild PASI score. **b** Keratin 10 vs. mild PASI score. **c** Keratin 16 vs. mild PASI score. **d** Keratin 17 vs. mild PASI score. **e** Keratin 14 vs. moderate PASI score. **f** Keratin 10 vs. moderate PASI score. **g** Keratin 16 vs. moderate PASI score. **h** Keratin 17 vs. moderate PASI score. The correlation coefficient (*r*) and significant value (*P*) are given in this figure

Correlation analysis of keratins and PASI in two stages of psoriasis is shown in Fig. 8. There was a significant inverse correlation between normal epidermal keratins K14 and K10 and PASI score (*r* = − 0.998; *r* = − 0.996; *P* < 0.001), respectively, in mild psoriasis, whereas the abnormal hyperproliferative keratins K16 and K17 showed a positive correlation with PASI score (*r* = 0.999; *r* = 0.997; *P* < 0.001) in mild psoriasis. In moderate case, Spearman’s coefficient correlation analysis showed a negative correlation between K14 and K10 and PASI score (*r* = − 0.993; *r* = − 0.997; *P* < 0.001), respectively, and showed a strong positive correlation between K16 and K17 with PASI score, which was statistically significant (*r* = 0.998; *r* = 0.998; *P* < 0.001), respectively. We report that the degree of severity of the clinical phenotype has been directly linked to the position of the pathogenic mutation along the keratin polypeptide backbone.

## Discussion

The epidermal barrier is formed by keratinocytes contributing tight junctions and the cornified envelope [1]. Barrier dysfunction and cutaneous sensitization can give rise to chronic inflammatory disorders like atopic eczema and psoriasis. Keratin plays an important role in maintaining epidermal barrier as well as in the formation of tight junction and cornified envelope in skin [40]. However, mutations of keratins in psoriasis have not been fully investigated. In this present study, we have identified 18 mutations in the CDS region of K14 out of which nine mutations are slightly pathogenic variants in the psoriatic samples. Numerous mutations in K5 and K14 identified in EBS are dominant pathogenic mutations. There are a few cases of recessive mutations which nearly all lead to loss of function or a natural knockout in K14 [41–45]. Related to our result, studies showed that in EBS, the pathogenic mutations of K14 usually occur within two segments (1A and 2B) of the rod domain [46]. Cells expressing mutated K14 are more prone to stress damage and resistance to apoptosis [47, 48]. Thus, we propose that the mutation of K14 in psoriatic skin might lead to “unnatural” keratin pair; this will drastically alter the assembly of network filament as well as mechanical support in epidermal skin and also increase the degradation of keratin filaments by various stressors, all these leading to acanthosis in psoriasis.

Moreover, we have also identified 13 mutated sequences in keratin 10 protein. Of these, six sequences are considered as highly pathogenic, and the remaining seven sequences are considered as slightly pathogenic. Similar to our study, in epidermal hyperkeratosis, most of the pathogenic missense mutations of K10 usually occur within highly conserved regions of the α-helical rod domains and the non-helical HI domain [49, 50]. Reichelt et al. [51] reported that defects in keratin 10 gene may lead to hyperproliferation of basal cells, induction of c-Myc, cyclin D1, 14-3-3σ, K6, and K16. K10 is essential to control cell proliferation and also for the expression of filaggrin and cornified envelope proteins [52]. In epidermolytic ichthyosis (EI) patients, mutation of K10 proteins showed increased proliferation of suprabasal keratinocytes which leads to ichthyosiform lesions, suprabasal cytolysis, blister formation, and hyperkeratosis [53, 54]. Thus, the mutation in K10 might cause irregularly shaped pathognomonic keratin intermediate filament (KIF) clumps in suprabasal keratinocytes which disrupt the structural stability in the suprabasal keratinocytes and also increase cyclin D, cyclin E, and phosho-Akt levels, all of which drive to hyperproliferation of keratinocytes in psoriasis.

Rapid psoriasis recurrence or new morphologies have been reported after discontinuation of traditional therapy or biological [55–58]. Interestingly, psoriatic lesions preferentially appeared at sites of wound healing (Koebner phenomenon). These relapse and Koebner phenomenon in psoriasis might be due to the variants in hyperproliferative keratin genes. In order to reveal this, we studied K16 and K17 psoriatic sequences for mutational analysis. In this, we did not find any mutation and expression of these keratins in mild psoriasis. As disease progresses, we found a pathogenic variant of K16 and K17 in moderate psoriasis. Early studies showed that an inherited dominant mutation in K16 and K17 is causative for pachyonychia congenita (PC) [59–63]. Lessard et al. [64] showed that in PC, loss of K16 function in mice causes the development of prominent calluses on the plantar side of the front and hind paws, which significantly compromise mobility and eventually lead to overt loss of barrier properties and also deletion of K16-produced spontaneously arising palmoplantar keratoderma-like lesions in mice. However, some studies have reported that in the context of epidermal wound healing, K16 mainly promotes a reorganization of the cytoplasmic array of keratin filaments, an event that precedes the onset of keratinocyte migration into the wound site [65], and also, K17-associated activation process is believed to be essential in reepithelization of the injured area [66]. In contrast to wound healing, K17 and K16 expression in psoriasis failed to resolve the deregulated inflammatory response which leads to the persistent activation of keratinocytes; these responses might be due to the mutation in these genes. Thus, mutation in K16 and K17 in psoriasis might be one of the factors for disease severity and Koebner phenomenon, accompanied with early relapse even with therapy.

In this study, we showed increased K14 and K10 expression at protein and mRNA levels in mild psoriatic skin biopsies compared to nonlesional skin biopsies; in contrast to this, moderate lesional biopsy showed decreased expression of K14 and K10. Similar to our results, other studies also showed that in lesional biopsy, the level of K14 was considerably higher with downregulation of K10 compared with normal epidermis [11, 13, 67]. Also, we found that the hyperproliferative keratin K16 and K17 expressions were low in mild psoriasis; as diseases progress, these expressions were increased in moderate psoriasis compared to nonlesional. Consistent with our results, many authors also showed an upregulation of K6 and K16 [68, 69] in psoriatic epidermis. Similarly, a study by de Jong et al. [69] also showed the overexpression of K17 in psoriatic epidermis. Earlier reports said that the K17 is considered as a therapeutic target and marker of antipsoriatic therapies used for the treatment of psoriasis [70, 71]. These finding suggests that the changes in keratin expressions might trigger and exacerbate psoriasis.

Finally, we also found a negative correlation between epidermal keratins and PASI score and positive correlation between hyperproliferative keratins and PASI score; these further added that alteration in these keratins in skin might cause severity of psoriasis. Previous studies have reported that the degree of severity of the clinical phenotype has been directly linked to the position of the pathogenic mutation along the keratin polypeptide backbone, although more recent reports provide some exceptions to this, whereby also milder disease phenotypes are caused by pathogenic mutations in the conserved hot spot region of the keratin genes [72, 73]. We demonstrate that the novel mutation of keratins found in psoriasis patients after comparison with ExAC database will be considered as efficient filtering of candidate psoriasis-causing variants and are useful for the discovery of human “knockout” psoriasis variants in protein-coding genes. Overall, we strongly believed that these novel keratin mutations in the psoriatic epidermis might be one of the main causative factors for psoriasis.

## Conclusion

Even though there are many highly psoriasis-risk genes identified, in 2009, GWAS have found the importance of keratin pattern in psoriasis. Also, studies have found that keratin plays a critical role in the pathogenesis of psoriasis. Based on all these aspects, we focused on this first and foremost study in which we have identified mutation in K14, K10, K16, and K17 genes in the two stages of psoriasis. Our findings suggest that this mutation leads to decreased expression of highly regulated keratins which in turn causes defective barrier accompanied with acanthosis, hyperplasia, and infiltration of inflammatory mediators in psoriatic epidermis. These findings will offer a clue to better understanding the pathogenesis of psoriasis.

## Additional files

Additional file 1: **Figure S1.** Comparison of the identified K14 and K10 nucleotide sequences of mild psoriasis obtained in this study with those of respective NCBI CDS sequences using BioEdit software (version 7.2). The mutated sequences are indicated by red arrows. **Figure S2.** Comparison of the identified K14, K10, K16, and K17 nucleotide sequences of moderate psoriasis obtained in this study with those of respective NCBI CDS sequences using BioEdit software (version 7.2). The mutated sequences are indicated by red arrows. (PDF 11608 kb)

Additional file 2: **Table S1.** K14, K10, K16, and K17 missense mutated variants retrieved from ExAC database. The mutated sequences identical both in our study and ExAC database are indicated by boldfaced words. (XLSX 54 kb)

## Abbreviations

CDS: Coding sequences
EBS: Epidermolysis bullosa simplex
EI: Epidermolytic ichthyosis
GWAS: Genome-wide association studies
IF: Intermediate filament
K10: Keratin 10
K14: Keratin 14
K16: Keratin 16
K17: Keratin 17
KIF: Keratin intermediate filament
PC: Pachyonychia congenita
SNP: Single nucleotide polymorphism
