# Supplementary material for: Mutational analysis of epidermal and hyperproliferative type I keratins in mild and moderate psoriasis vulgaris patients: a possible role in the pathogenesis of psoriasis along with disease severity
Source: Hum Genomics. 2018 May 21;12:27. doi: 10.1186/s40246-018-0158-2 (PMC5963134; doi:10.1186/s40246-018-0158-2)
Supplement: Supplementary file 1 — Figure S1. Comparison of the identified K14 and K10 nucleotide sequences of mild psoriasis obtained in this study with those of respective NCBI CDS sequences using BioEdit software (version 7.2). The mutated sequences are indicated by red arrows. Figure S2. Comparison of the identified K14, K10, K16, and K17 nucleotide sequences of moderate psoriasis obtained in this study with those of respective NCBI CDS sequences using BioEdit software (version 7.2). The mutated sequences are indicated by red arrows. (PDF 11608 kb) [file 40246_2018_158_MOESM1_ESM.pdf]

Figure S1: Comparison of the identified epidermal type I keratin nucleotide sequence of mild psoriasis obtained in this study with those of respective NCBI CDS sequences. The mutated sequences are indicated by arrow.

```
KRT14                 1     ATGACCACCTGCAGCCGCCAGTTCACCTCCTCCAGCTCCATGAAGGGCTCCTGCGGCATCGGGGGCGGCATCGGGGGCGGCTCCAGCCGCATCTCCTCCG  100
Mild Psoriatic KRT14  1     ATGACCACCTGCAGCCGCCAGTTCACCTCCTCCAGCTCCATGAAGGGCTCCTGCGGCATCGGGGGCGGCATCGGGGGCGGCTCCAGCCGCATCTCCTCCG  100

KRT14                 101   TCCTGGCCGGAGGGTCCTGCCGCGCCCCCAGCACCTACGGGGGCGGCCTGTCTGTCTCATCCTCCCGCTTCTCCTCTGGGGGAGCCTACGGGCTGGGGGG  200
Mild Psoriatic KRT14  101   TCCTGGCCGGAGGGTCCTGCCGCGCCCCCAGCACCTACGGGGGCGGCCTGTCTGTCTCATCCTCCCGCTTCTCCTCTGGGGGAGCCTACGGGCTGGGGGG  200

KRT14                 201   CGGCTATGGCGGTGGCTTCAGCAGCAGCAGCAGCAGCTTTGGTAGTGGCTTTGGGGGAGGATATGGTGGTGGCCTTGGTGCTGGCTTGGGTGGTGGCTTT  300
Mild Psoriatic KRT14  201   CGGCTATGGCGGTGGCTTCAGCAGCAGCAGCAGCAGCTTTGGTAGTGGCTTTGGGGGAGGATATGGTGGTGGCCTTGGTGCTGGCTTGGGTGGTGGCTTT  300

KRT14                 301   GGTGGTGGCTTTGCTGGTGGTGATGGGCTTCTGGTGGGCAGTGAGAAGGTGACCATGCAGAACCTCAATGACCGCCTGGCCTCCTACCTGGACAAGGTGC  400
Mild Psoriatic KRT14  301   GGTGGTGGCTTTGCTGGTGGTGATGGGCTTCTGGTGGGCAGTGAGAAGGTGACCATGCAGAACCTCAATGAACGCCTGGCCTCCTACCTGGACAAGGTGC  400

KRT14                 401   GTGCTCTGGAGGAGGCCAACGCCGACCTGGAAGTGAAGATCCGTGACTGGTACCAGAGGCAGCGGCCTGCTGAGATCAAAGACTACAGTCCCTACTTCAA  500
Mild Psoriatic KRT14  401   GTGCTCTGGAGGACGCCAACGCCGACCTGGAAGTGAAGATCCGTGACTGGTACCAGAGGCAGCGGCTTGCTGAGATCATAGACTACAGTCCCTACTTCAA  500

KRT14                 501   GACCATTGAGGACCTGAGGAACAAGATTCTCACAGCCACAGTGGACAATGCCAATGTCCTTCTGCAGATTGACAATGCCCGTCTGGCCGCGGATGACTTC  600
Mild Psoriatic KRT14  501   GACCATTGAGGACCTGAGGAACAAGATTCTCACAGCCACAGTGGACAATGCCAATGTCCTTCTGCAGATTGACAATGCCCGTCTGGCCGCGGATGACTTC  600

KRT14                 601   CGCACCAAGTATGAGACAGAGTTGAACCTGCGCATGAGTGTGGAAGCCGACATCAATGGCCTGCGCAGGGTGCTGGACGAACTGACCCTGGCCAGAGCTG  700
Mild Psoriatic KRT14  601   CGCACCAAGTATGAGACAGAGTTGAACCTGCGCATGAGTGTGGAAGCCGACATCAATGTCCTGCGCAGGGTGCTGGACGAACTGACCCTGGCCAGAGCTG  700

KRT14                 701   ACCTGGAGATGCAGATTGAGAGCCTGAAGGAGGAGCTGGCCTACCTGAAGAAGAACCACGAGGAGGAGATGAATGCCCTGAGAGGCCAGGTGGGTGGAGA  800
Mild Psoriatic KRT14  701   ACCTGGAGATGCAGATTGAGAGCCTGAAGGAGGAGCTGGCCTACCTGAAGAAGAACCACGAGGAGGAGATGAATGCCCTGAGAGGCCAGGTGGGTGGAGA  800

KRT14                 801   TGTCAATGTGGAGATGGACGCTGCACCTGGCGTGGACCTGAGCCGCATTCTGAACGAGATGCGTGACCAGTATGAGAAGATGGCAGAGAAGAACCGCAAG  900
Mild Psoriatic KRT14  801   TGTCAATGTGGAGATGGACGCTGCACCTGGCGTGGACCTGAGCCGCATTCTGAACGAGATGCGTGACCAGTATGAGAAGATGGCAGAGAAGAACCCCAAG  900

KRT14                 901   GATGCCGAGGAATGGTTCTTCACCAAGACAGAGGAGCTGAACCGCGAGGTGGCCACCAACAGCGAGCTGGTGCAGAGCGGCAAGAGCGAGATCTCGGAGC  1000
Mild Psoriatic KRT14  901   GATGCCGAGGAATGGTTCTTCACCAAGACAGAGGAGCTGAACCCCGAGGTGGCCACCAACAGCGAGCTGGTGCAGAGCGGCAAGAGCGAGATCTCGGAGC  1000

KRT14                 1001  TCCGGCGCACCATGCAGAACCTGGAGATTGAGCTGCAGTCCCAGCTCAGCATGAAAGCATCCCTGGAGAACAGCCTGGAGGAGACCAAAGGTCGCTACTG  1100
Mild Psoriatic KRT14  1001  TCCGGCGCACCATGCAGAACCTGGAGATTGAGCTGCAGTCCCAGCTCAGCATGAAAGCATCCCTGGAGAACAGCCTGGAGGAGACCAAAGGTCGCTACTG  1100

KRT14                 1101  CATGCAGCTGGCCCAGATCCAGGAGATGATTGGCAGCGTGGAGGAGCAGCTGGCCCAGCTCCGCTGCGAGATGGAGCAGCAGAACCAGGAGTACAAGATC  1200
Mild Psoriatic KRT14  1101  CATGCAGCTGGCCCAGATCCAGGAGATGATTGGCAGCGTGGAGGAGCAGCTGGCCCAGCTCCGCTGCGAGATGGAGCAGCAGAACCAGGAGTACAAGATC  1200

KRT14                 1201  CTGCTGGACGTGAAGACGCGGCTGGAGCAGGAGATCGCCACCTACCGCCGCCTGCTGGAGGGCGAGGACGCCCACCTCTCCTCCTCCCAGTTCTCCTCTG  1300
Mild Psoriatic KRT14  1201  CTGCTGGACGTGAAGACGCGGCTGGAGCAGGAGATCACCACCTACTGCCGCCTGCTGGAGGGCGAGGACGCCCACCTCTCCTCCTCCCAGTTCTCCTCTG  1300

KRT14                 1301  GATCGCAGTCATCCAGAGATGTGACCTCCTCCAGCCGCCAAATCCGCACCAAGGTCATGGATGTGCACGATGGCAAGGTGGTGTCCACCCACGAGCAGGT  1400
Mild Psoriatic KRT14  1301  GATCGCAGTCATCCAGAGATGTGACCTCCTCCAGCCGCCAAATCCGCACCAAGGTCATGGATGTGCACGATGGCAAGGTGGTGTCCACCCACGAGCAGGT  1400

KRT14                 1401  CCTTCGCACCAAGAACTGA  1419
Mild Psoriatic KRT14  1401  CCTTCGCACCAAGAACTGA  1419
```

Figure S1: Comparison of the identified epidermal type I keratin nucleotide sequence of mild psoriasis obtained in this study with those of respective NCBI CDS sequences. The mutated sequences are indicated by arrow.

**KRT10**	1	ATGTCTGTTCGATACAGCTCAAGCAAGCACTACTCTTCCTCCCGCAGTGGAGGAGGAGGAGGAGGAGGAGGATGTGGAGGAGGAGGAGGAGTGTCATCCC	100
**Mild Psoriatic KRT10**	1	ATGTCTGTTCGATACAGCTCAAGCAAGCACTACTCTTCCTCCCGCAGTGGAGGAGGAGGAGGAGGAGGAGGATGTGGAGGAGGAGGAGGAGTGTCATCCC	100
**KRT10**	101	TAAGAATTTCTAGCAGCAAAGGCTCCCTTGGTGGAGGATTTAGCTCAGGGGGGTTCAGTGGTGGCTCTTTTAGCCGTGGGAGCTCTGGTGGGGGCTGCTT	200
**Mild Psoriatic KRT10**	101	TAAGAATTTCTAGCAGCAAAGGCTCCCTTGGTGGAGGATTTAGCTCAGGGGGGTTCAGTGGTGGCTCTTTTAGCCGTGGGAGCTCTGGTGGGGGCTGCTT	200
**KRT10**	201	TGGGGGCTCATCAGGTGGCTATGGAGGATTAGGAGGTTTTGGTGGAGGTAGCTTTCGTGGAAGCTATGGAAGTAGCAGCTTTGGTGGGAGTTATGGAGGC	300
**Mild Psoriatic KRT10**	201	TGGGGGCTCATCAGGTGGCTATGGAGGATTAGGAGGTTTTGGTGGAGGTAGCTTTCGTGGAAGCTATGGAAGTAGCAGCTTTGGTGGGAGTTATGGAGGC	300
**KRT10**	301	AGCTTTGGAGGGGGCAGTTTCGGAGGTGGCAGCTTTGGTGGGGGCAGCTTTGGTGGAGGCGGCTTTGGTGGAGGCGGCTTTGGAGGAGGCTTTGGTGGTG	400
**Mild Psoriatic KRT10**	301	AGCTTTGGAGGGGGCAGTTTCGGAGGTGGCAGCTTTGGTGGGGGCAGCTTTGGTGGAGGCGGCTTTGGTGGAGGCGGCTTTGGAGGAGGCTTTGGTGGTG	400
**KRT10**	401	GATTTGGAGGAGATGGTGGCCTTCTCTCTGGAAATGAAAAAGTAACCATGCAGAATCTGAATGACCGCCTGGCTTCCTACTTGGACAAAGTTCGGGCTCT	500
**Mild Psoriatic KRT10**	401	GATTTGGAGGAGAAGGTGGCCTTCTCTTTGGAAATGAAAAAGTAACCATGCAGAATCTGAATGACCGCCTGGCTTCCTACTTGGACAAAGTTCGGGCTCT	500
**KRT10**	501	GGAAGAATCAAACTATGAGCTGGAAGGCAAAATCAAGGAGTGGTATGAAAAGCATGGCAACTCACATCAGGGGGAGCCTCGTGACTACAGCAAATACTAC	600
**Mild Psoriatic KRT10**	501	GCAAGAATCAAACTATGAGCTGGAAGGCAAAATCAAGGAGTGGTATGAAAAGCATGGCAACTCACATCAGGGGGAGCCTCGTGACTACAGCAAATACTAC	600
**KRT10**	601	AAAACCATCGATGACCTTAAAAATCAGATTCTCAACCTAACAACTGATAATGCCAACATCCTGCTTCAGATCGACAATGCCAGGCTGGCAGCTGATGACT	700
**Mild Psoriatic KRT10**	601	AAAACCATCGATGACCTTAAAAATCAGATTCTCAACCTAACAACTGATAATGCCAACATCCTGCTTCAGATCGACAATTCCAGGCTGGCAGCTGATGACT	700
**KRT10**	701	TCAGGCTGAAGTATGAGAATGAGGTAGCTCTGCGCCAGAGCGTGGAGGCTGACATCAACGGCCTGCGTAGGGTGCTGGATGAGCTGACCCTGACCAAGGC	800
**Mild Psoriatic KRT10**	701	TCAGGCTGAAGTATCAGAATGAGGTAGCTGTGCGCCAGAGCGTGGAGGCTGACATCAACGGCCTGCGTATGGTGCTGGATGAGCTGACCCTGACCAAGGC	800
**KRT10**	801	TGACCTGGAGATGCAAATTGAGAGCCTGACTGAAGAGCTGGCCTATCTGAAGAAGAACCACGAGGAGGAAATGAAAGACCTTCGAAATGTGTCCACTGGT	900
**Mild Psoriatic KRT10**	801	TGACCTGGAGATGCAAATTGAGAGCCTGACTGAAGAGCTGGCCTATCTGAAGAAGAACCACGAGGAGGAAATGAAAGACCTTCGAAATGTGTCCACTGGT	900
**KRT10**	901	GATGTGAATGTGGAAATGAATGCTGCCCCGGGTGTTGATCTGACTCAACTTCTGAATAACATGAGAAGCCAATATGAACAACTTGCTGAACAAAACCGCA	1000
**Mild Psoriatic KRT10**	901	GATGTGAATGTGGAAATGAATGCTGCCCCGGGTGTTGATCTGACTCAACTTCTGAATAACATGAGAAGCCAATATGAACAACTTGCTGAACAAAACCGCA	1000
**KRT10**	1001	AAGATGCTGAAGCCTGGTTCAATGAAAAGAGCAAGGAACTGACTACAGAAATTGATAATAACATTGAACAGATATCCAGCTATAAATCTGAGATTACTGA	1100
**Mild Psoriatic KRT10**	1001	AAGATGCTGAAGCCTGGTTCAATGAAAAGAGCAAGGAACTGACTACAGAAATTGATAATAACATTGAACAGATATCCAGCTATAAATCTGAGATTACTGA	1100
**KRT10**	1101	ATTGAGACGTAATGTACAAGCTCTGGAGATAGAACTACAGTCCCAACTGGCCTTGAAACAATCCCTGGAAGCCTCCTTGGCAGAAACAGAAGGTCGCTAC	1200
**Mild Psoriatic KRT10**	1101	ATTGAGACGTAATGTACAAGCTCTGGAGATAGAACTACAGTCCCAACTGGCCTTGAAACAATCCCTGGAAGCCTCCTTGGCAGAAACAGAAGGTCGCTAC	1200
**KRT10**	1201	TGTGTGCAGCTCTCACAGATTCAGGCCCAGATATCCGCTCTGGAAGAACAGTTGCAACAGATTCGAGCTGAAACCGAGTGCCAGAATACTGAATACCAAC	1300
**Mild Psoriatic KRT10**	1201	TGTGTGCAGCTCTCACAGATTCAGGCCCAGATATCCGCTCTGGAAGAACAGTTGCAACAGATTCGAGCTGAAACCGAGTGCCAGAATACTGAATACCAAC	1300
**KRT10**	1301	AACTCCTGGATATTAAGATCCGACTGGAGAATGAAATTCAAACCTACCGCAGCCTGCTAGAAGGAGAGGGAAGTTCCGGAGGCGGCGGACGCGGCGGCGG	1400
**Mild Psoriatic KRT10**	1301	AACTCCTGGATATTAAGATCCGACTGGAGAATGAAATTCAAACCTACCGCAGCCTGCTAGAAGGAGAGGGAAGTTCCGGAGGCGGCGGACGCGGCGGCGG	1400
**KRT10**	1401	AAGTTTCGGCGGCGGCTACGGCGGCGGAAGCTCCGGCGGCGGAAGCTCCGGCGGCGGCCACGGCGGCGGCCACGGCGGCAGTTCCGGCGGCGGCTACGGA	1500
**Mild Psoriatic KRT10**	1401	AAGTTTCGGCGGCGGCTACGGCGGCGGAAGCTCCGGCGGCGGAAGCTCCGGCGGCGGCCACGGCGGCGGCCACGGCGGCAGTTCCGGCGGCGGCTACGGA	1500
**KRT10**	1501	GGCGGAAGCTCCGGCGGCGGAAGCTCCGGCGGCGGCTACGGGGGCGGAAGCTCCAGCGGCGGCCACGGCGGCAGTTCCAGCGGCGGCTACGGTGGTGGCA	1600
**Mild Psoriatic KRT10**	1501	GGCGGAAGCTCCGGCGGCGGAAGCTCCGGCGGCGGCTACGGGGGCGGAAGCTCCAGCGGCGGCCACGGCGGCAGTTCCAGCGGCGGCTACGGTGGTGGCA	1600
**KRT10**	1601	GTTCCGGCGGCGGCGGCGGCGGCTACGGGGGCGGCAGCTCCGGCGGCGGCAGCAGCTCCGGCGGCGGATACGGCGGCGGCAGCTCCAGCGGAGGCCACAA	1700
**Mild Psoriatic KRT10**	1601	GTTCCGGCGGCGGCGGCGGCGGCTACGGGGGCGGCAGCTCCGGCGGCGGCAGCAGCTCCGGCGGCGGATACGGCGGCGGCAGCTCCAGCGGAGGCCACAA	1700
**KRT10**	1701	GTCCTCCTCTTCCGGGTCCGTGGGCGAGTCTTCATCTAAGGGACCAAGATACTAA	1755
**Mild Psoriatic KRT10**	1701	GTCCTCCTCTTCCGGGTCCGTGGGCGAGTCTTCATCTAAGGGACCAAGATACTAA	1755

Figure S2: Comparison of the identified epidermal and hyperproliferative type I keratin nucleotide sequence of moderate psoriasis obtained in this study with those of respective NCBI CDS sequences. The mutated sequences are indicated by arrow.

```
KRT14                     1     ATGACCACCTGCAGCCGCCAGTTCACCTCCTCCAGCTCCATGAAGGGCTCCTGCGGCATCGGGGGCGGCATCGGGGGCGGCTCCAGCCGCATCTCCTCCG   100
Moderate Psoriatic KRT14  1     ATGACCACCTGCAGCCGCCAGTTCACCTCCTCCAGCTCCATGAAGGGCTCCTGCGGCATCGGGGGCGGCATCGGGGGCGGCTCCAGCCGCATCTCCTCCG   100

KRT14                     101   TCCTGGCCGGAGGGTCCTGCCGCGCCCCCAGCACCTACGGGGGCGGCCTGTCTGTCTCATCCTCCCGCTTCTCCTCTGGGGGAGCCTACGGGCTGGGGGG   200
Moderate Psoriatic KRT14  101   TCCTGGCCGGAGGGTCCTGCCGCGCCCCCAGCACCTACGGGGGCGGCCTGTCTGTCTCATCCTCCCGCTTCTCCTCTGGGGGAGCCTACGGGCTGGGGGG   200

KRT14                     201   CGGCTATGGCGGTGGCTTCAGCAGCAGCAGCAGCAGCTTTGGTAGTGGCTTTGGGGGAGGATATGGTGGTGGCCTTGGTGCTGGCTTGGGTGGTGGCTTT   300
Moderate Psoriatic KRT14  201   CGGCTATGGCGGTGGCTTCAGCAGCAGCAGCAGCAGCTTTGGTAGTGGCTTTGGGGGAGGATATGGTGGTGGCCTTGGTGCTGGCTTGGGTGGTGGCTTT   300

KRT14                     301   GGTGGTGGCTTTGCTGGTGGTGATGGGCTTCTGGTGGGCAGTGAGAAGGTGACCATGCAGAACCTCAATGACCGCCTGGCCTCCTACCTGGACAAGGTGC   400
Moderate Psoriatic KRT14  301   GGTGGTGGCTTTGCTGGTGGTGATGGGCTTCTGGTGGGCAGTGAGAAGGTGACCATGCAGAACCTCAATGAACGCCTGGCCTCCTACGTGGACAAGGTGC   400

KRT14                     401   GTGCTCTGGAGGAGGCCAACGCCGACCTGGAAGTGAAGATCCGTGACTGGTACCAGAGGCAGCGGCCTGCTGAGATCAAAGACTACAGTCCCTACTTCAA   500
Moderate Psoriatic KRT14  401   GTGCTCTGGAGGACCCCATCGCCGACCTGGAAGTGAAGATCTGTGACTGGTACCAGAGGCAGCGGCTTGCTGAGATCATAGACTACAGTCCCTACTTCAA   500

KRT14                     501   GACCATTGAGGACCTGAGGAACAAGATTCTCACAGCCACAGTGGACAATGCCAATGTCCTTCTGCAGATTGACAATGCCCGTCTGGCCGCGGATGACTTC   600
Moderate Psoriatic KRT14  501   GACCATTGAGGTCCTGAGTAACAAGATTCTCACAGCCACAGTGGACAATGCCAATGTCCTTCTGCAGATTGACAATGCCCGTCTGGCCGCGGATGACTTC   600

KRT14                     601   CGCACCAAGTATGAGACAGAGTTGAACCTGCGCATGAGTGTGGAAGCCGACATCAATGGCCTGCGCAGGGTGCTGGACGAACTGACCCTGGCCAGAGCTG   700
Moderate Psoriatic KRT14  601   CGCACCAAGTATGAGACAGAGTTGAACCTGCGCATGAGTGTGGAAGCCGACATCAATGTCCTGCGCAGGGTGCTGGACGAACTGACCCTGGCCAGAGCTG   700

KRT14                     701   ACCTGGAGATGCAGATTGAGAGCCTGAAGGAGGAGCTGGCCTACCTGAAGAAGAACCACGAGGAGGAGATGAATGCCCTGAGAGGCCAGGTGGGTGGAGA   800
Moderate Psoriatic KRT14  701   ACCTGGAGATGCAGATTGAGAGCCTGAAGGAGGAGCTGGCCTACCTGAAGAAGAACCACGAGGAGGAGATGAATGCCCTGAGAGGCCAGGTGGGTGGAGA   800

KRT14                     801   TGTCAATGTGGAGATGGACGCTGCACCTGGCGTGGACCTGAGCCGCATTCTGAACGAGATGCGTGACCAGTATGAGAAGATGGCAGAGAAGAACCGCAAG   900
Moderate Psoriatic KRT14  801   TGTCAATGTGGAGATGGACGCTGCACCTGGCGTGGACCTGAGCCGCATTCTGAACGAGATGCGTGACCAGTATGAGAAGATGGCAGAGAAGAACCCCAAG   900

KRT14                     901   GATGCCGAGGAATGGTTCTTCACCAAGACAGAGGAGCTGAACCGCGAGGTGGCCACCAACAGCGAGCTGGTGCAGAGCGGCAAGAGCGAGATCTCGGAGC   1000
Moderate Psoriatic KRT14  901   GATCCCGAGGAATGGTTCTTCACCAAGACAGAGGAGCTGAACCCCGAGGTGGCCACCAACAGCGAGCTGGTGCAGAGCGGCAAGAGCGAGATCTCGGAGC   1000

KRT14                     1001  TCCGGCGCACCATGCAGAACCTGGAGATTGAGCTGCAGTCCCAGCTCAGCATGAAAGCATCCCTGGAGAACAGCCTGGAGGAGACCAAAGGTCGCTACTG   1100
Moderate Psoriatic KRT14  1001  TCCGGCGCACCATGCAGAACCTGGAGATTGAGCTGCAGTCCCAGCTCAGCATGAAACCATCCCTGGAGAACAGCCTGGAGGAGACCAAAGGTCGCTACTG   1100

KRT14                     1101  CATGCAGCTGGCCCAGATCCAGGAGATGATTGGCAGCGTGGAGGAGCAGCTGGCCCAGCTCCGCTGCGAGATGGAGCAGCAGAACCAGGAGTACAAGATC   1200
Moderate Psoriatic KRT14  1101  CATGCAGCTGGCCCAGATCCAGGAGATGATTGGCAGCGTGGAGGAGCAGCTGGCCCAGCTCCGCTGCGAGATGGAGCAGCAGAACCAGGAGTACAAGATC   1200

KRT14                     1201  CTGCTGGACGTGAAGACGCGGCTGGAGCAGGAGATCGCCACCTACCGCCGCCTGCTGGAGGGCGAGGACGCCCACCTCTCCTCCTCCCAGTTCTCCTCTG   1300
Moderate Psoriatic KRT14  1201  CTGCTGGACGTGAAGACGCGGCTGGAGCAGGAGTTCACCACCTACTGCCGCCTGCTGGAGGGCGAGGACGCCCACCTCTCCTCCTCCCAGTTCTCCTCTG   1300

KRT14                     1301  GATCGCAGTCATCCAGAGATGTGACCTCCTCCAGCCGCCAAATCCGCACCAAGGTCATGGATGTGCACGATGGCAAGGTGGTGTCCACCCACGAGCAGGT   1400
Moderate Psoriatic KRT14  1301  GATCGCAGTCATCCAGAGATGTGACCTCCTCCAGCCGCCAAATCCGCACCAAGGTCATGGATGTGCACGATGGCAAGGTGGTGTCCACCCACGAGCAGGT   1400

KRT14                     1401  CCTTCGCACCAAGAACTGA   1419
Moderate Psoriatic KRT14  1401  CCTTCGCACCAAGAACTGA   1419
```

Figure S2: Comparison of the identified epidermal and hyperproliferative type I keratin nucleotide sequence of moderate psoriasis obtained in this study with those of respective NCBI CDS sequences. The mutated sequences are indicated by arrow.

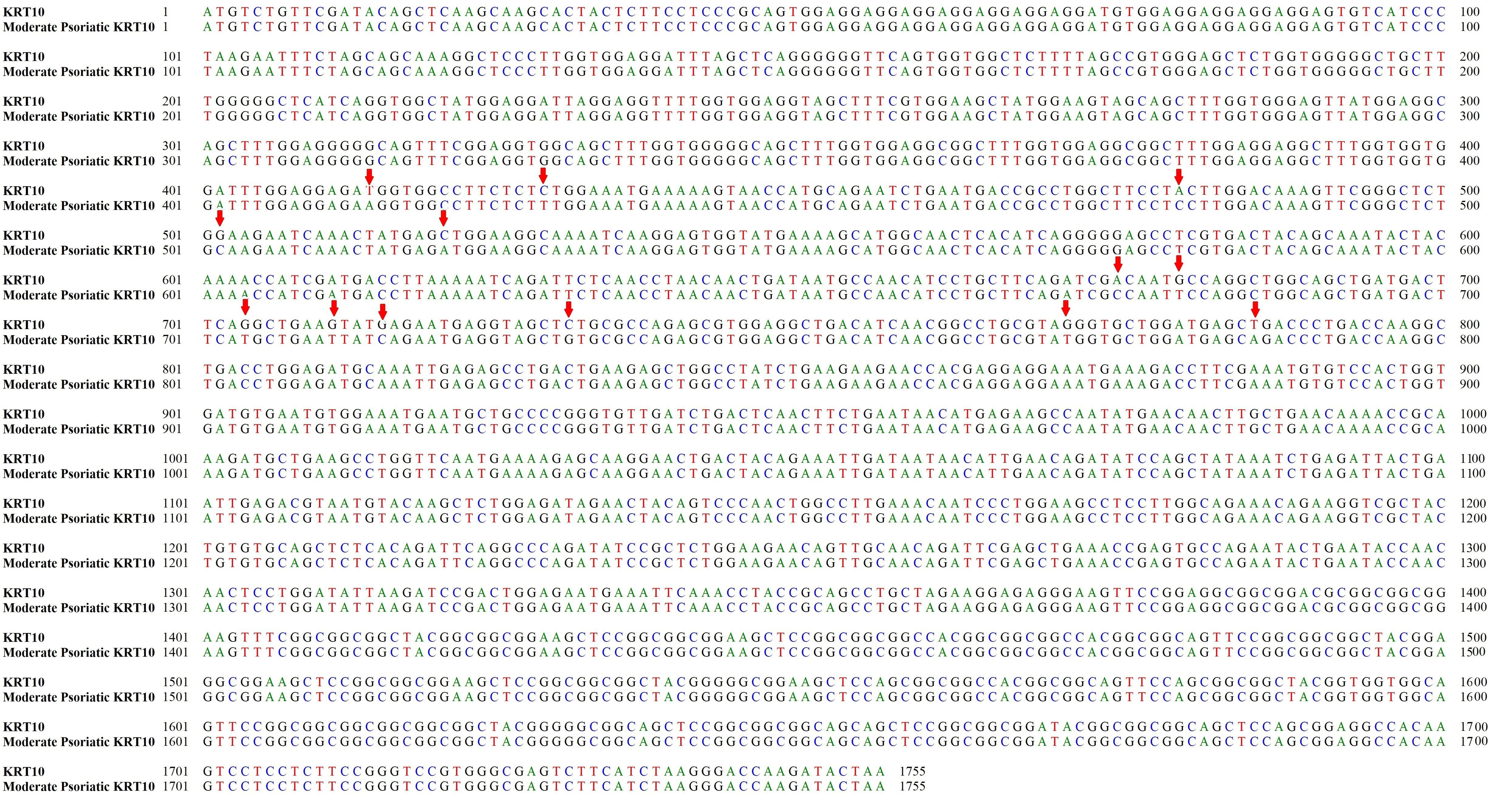

Figure S2: Comparison of the identified epidermal and hyperproliferative type I keratin nucleotide sequence of moderate psoriasis obtained in this study with those of respective NCBI CDS sequences. The mutated sequences are indicated by arrow.

**KRT16**	1	ATGACCACCTGCAGCCGCCAGTTCACCTCCTCCAGCTCCATGAAGGGCTCCTGCGGCATCGGAGGCGGCATCGGGGGCGGCTCCAGCCGCATCTCCTCCG	100
**Moderate Psoriatic KRT16**	1	ATGACCACCTGCAGCCGCCAGTTCACCTCCTCCAGCTCCATGAAGGGCTCCTGCGGCATCGGAGGCGGCATCGGGGGCGGCTCCAGCCGCATCTCCTCCG	100
**KRT16**	101	TCCTGGCCGGAGGGTCCTGCCGTGCCCCCAGCACCTACGGGGGCGGCCTGTCTGTCTCCTCTCGCTTCTCCTCTGGGGGAGCCTGCGGGCTGGGGGGCGG	200
**Moderate Psoriatic KRT16**	101	TCCTGGCCGGAGGGTCCTGCCGTGCCCCCAGCACCTACGGGGGCGGCCTGTCTGTCTCCTCTCGCTTCTCCTCTGGGGGAGCCTGCGGGCTGGGGGGCGG	200
**KRT16**	201	CTATGGCGGTGGCTTCAGCAGCAGCAGCTTTGGTAGTGGCTTCGGGGGAGGATATGGTGGTGGCCTTGGTGCTGGCTTCGGTGGTGGCTTGGGTGCT	300
**Moderate Psoriatic KRT16**	201	CTATGGCGGTGGCTTCAGCAGCAGCAGCTTTGGTAGTGGCTTCGGGGGAGGATATGGTGGTGGCCTTGGTGCTGGCTTCGGTGGTGGCTTGGGTGCT	300
**KRT16**	301	GGCTTTGGTGGTGGTTTTGCTGGTGGTGATGGGCTTCTGGTGGGCAGTGAGAAGGTGACCATGCAGAACCTCAATGACCGCCTGGCCTCCTACCTGGACA	400
**Moderate Psoriatic KRT16**	301	GGCTTTGGTGGTGGTTTTGCTGGTGGTGATGGGCTTCTGGTGGGCAGTGAGAAGGTGACCATGCAGAACCTCAATGACCGCCTGGCCTCCTACCTGGACA	400
**KRT16**	401	AGGTGCGTGCTCTGGAGGAGGCCAACGCCGACCTGGAAGTGAAGATCCGTGACTGGTACCAGAGGCAGCGGCCCAGTGAGATCAAAGACTACAGTCCCTA	500
**Moderate Psoriatic KRT16**	401	AGGTGCGTGTTCTGGAGGAGGCCAACGCCGACCTGGAAGTGAAGATCCGTGACTGGTACCAGAGGCAGCGGCCCAGTGAGATCAAAGACTACAGTCCCTA	500
**KRT16**	501	CTTCAAGACCATCGAGGACCTGAGGAACAAGATCATTGCGGCCACCATTGAGAATGCGCAGCCCATTTTGCAGATTGACAATGCCAGGCTGGCAGCCGAT	600
**Moderate Psoriatic KRT16**	501	CTTCAAGACCATCGAGGACCTGAGGAACAAGATCATTGCGGCCACCATTGAGAATGCGCAGCCCATTTTGCAGATTGACAATTCCAGGATGGCAGCCGAT	600
**KRT16**	601	GACTTCAGGACCAAGTATGAGCATGAACTGGCCCTGCGGCAGACTGTGGAGGCCGACGTCAATGGCCTGCGCCGGGTGTTGGATGAGCTGACCCTGGCCA	700
**Moderate Psoriatic KRT16**	601	GACTTCAGGACCAAGTATGAGCATGAACTGGCCGTGCGGCAGACTGTGGAGGCCGACGTCAATGGCGTGCGCCGGGTGTTGGATGAGCTGACCCTGGCCA	700
**KRT16**	701	GGACTGACCTGGAGATGCAGATCGAAGGCCTGAAGGAGGAGCTGGCCTACCTGAGGAAGAACCACGAGGAGGAGATGCTTGCTCTGAGAGGTCAGACCGG	800
**Moderate Psoriatic KRT16**	701	GGACTGACCTGGAGATGCAGATCGAAGGCCTGAAGGAGGAGCTGGCCTACCTGAGGAAGAACCACGAGGAGGAGATGCTTGCTCTGAGAGGTCAGACCGG	800
**KRT16**	801	CGGAGATGTGAACGTGGAGATGGATGCTGCACCTGGCGTGGACCTGAGCCGCATCCTGAATGAGATGCGTGACCAGTACGAGCAGATGGCAGAGAAAAAC	900
**Moderate Psoriatic KRT16**	801	CGGAGATGTGAACGTGGAGATGGATGCTGCACCTGGCGTGGACCTGAGCCGCATCCTGAATGAGATGCGTGACCAGTACGAGCAGATGGCAGAGAAAAAC	900
**KRT16**	901	CGCAGAGACGCTGAGACCTGGTTCCTGAGCAAGACCGAGGAGCTGAACAAAGAAGTGGCCTCCAACAGCGAACTGGTACAGAGCAGCCGCAGTGAGGTGA	1000
**Moderate Psoriatic KRT16**	901	CGCAGAGACGCTGAGACCTGGTTCCTGAGCAAGACCGAGGAGCTGAACAAAGAAGTGGCCTCCAACAGCGAACTGGTACAGAGCAGCCGCAGTGAGGTGA	1000
**KRT16**	1001	CGGAGCTCCGGAGGGTGCTCCAGGGCCTGGAGATTGAGCTGCAGTCCCAGCTCAGCATGAAAGCATCCCTGGAGAACAGCCTGGAGGAGACCAAAGGCCG	1100
**Moderate Psoriatic KRT16**	1001	CGGAGCTCCGGAGGGTGCTCCAGGGCCTGGAGATTGAGCTGCAGTCCCAGCTCAGCATGAAAGCATCCCTGGAGAACAGCCTGGAGGAGACCAAAGGCCG	1100
**KRT16**	1101	CTACTGCATGCAGCTGTCCCAGATCCAGGGACTGATTGGCAGTGTGGAGGAGCAGCTGGCCCAGCTACGCTGTGAGATGGAGCAGCAGAGCCAGGAGTAC	1200
**Moderate Psoriatic KRT16**	1101	CTACTGCATGCAGCTGTCCCAGATCCAGGGACTGATTGGCAGTGTGGAGGAGCAGCTGGCCCAGCTACGCTGTGAGATGGAGCAGCAGAGCCAGGAGTAC	1200
**KRT16**	1201	CAGATCTTGCTGGATGTGAAGACGCGGCTGGAGCAGGAGATTGCCACCTACCGCCGCCTGCTGGAGGGCGAGGATGCCCACCTTTCCTCCCAGCAAGCAT	1300
**Moderate Psoriatic KRT16**	1201	CAGATCTTGCTGGATGTGAAGACGCGGCTGGAGCAGGAGATTGCCACCTACCGCCGCCTGCTGGAGGGCGAGGATGCCCACCTTTCCTCCCAGCAAGCAT	1300
**KRT16**	1301	CTGGCCAATCCTATTCTTCCCGCGAGGTCTTCACCTCCTCCTCGTCCTCTTCGAGCCGTCAGACCCGGCCCATCCTCAAGGAGCAGAGCTCATCCAGCTT	1400
**Moderate Psoriatic KRT16**	1301	CTGGCCAATCCTATTCTTCCCGCGAGGTCTTCACCTCCTCCTCGTCCTCTTCGAGCCGTCAGACCCGGCCCATCCTCAAGGAGCAGAGCTCATCCAGCTT	1400
**KRT16**	1401	CAGCCAGGGCCAGAGCTCCTAG	1422
**Moderate Psoriatic KRT16**	1401	CAGCCAGGGCCAGAGCTCCTAG	1422

Figure S2: Comparison of the identified epidermal and hyperproliferative type I keratin nucleotide sequence of moderate psoriasis obtained in this study with those of respective NCBI CDS sequences. The mutated sequences are indicated by arrow.

```
KRT 17                   1     ATGACCACCTCCATCCGCCAGTTCACCTCCTCCAGCTCCATCAAGGGCTCCTCCGGCCTGGGGGGCGGCTCGTCCCGCACCTCCTGCCGGCTGTCTGGCG  100
Moderate Psoriatic KRT17 1     ATGACCACCTCCATCCGCCAGTTCACCTCCTCCAGCTCCATCAAGGGCTCCTCCGGCCTGGGGGGCGGCTCGTCCCGCACCTCCTGCCGGCTGTCTGGCG  100

KRT 17                   101   GCCTGGGTGCCGGCTCCTGCAGGCTGGGATCTGCTGGCGGCCTGGGCAGCACCCTCGGGGGTAGCAGCTACTCCAGCTGCTACAGCTTTGGCTCTGGTGG  200
Moderate Psoriatic KRT17 101   GCCTGGGTGCCGGCTCCTGCAGGCTGGGATCTGCTGGCGGCCTGGGCAGCACCCTCGGGGGTAGCAGCTACTCCAGCTGCTACAGCTTTGGCTCTGGTGG  200

KRT 17                   201   TGGCTATGGCAGCAGCTTTGGGGGTGTTGATGGGCTGCTGGCTGGAGGTGAGAAGGCCACCATGCAGAACCTCAATGACCGCCTGGCCTCCTACCTGGAC  300
Moderate Psoriatic KRT17 201   TGGCTATGGCAGCAGCTTTGGGGGTGTTGATGGGCTGCTGGCTGGAGGTGAGAAGGCCACCATGCAGAACCTCAATGACCGCCTGGCCTCCTACCTGGAC  300

KRT 17                   301   AAGGTGCGTGCCCTGGAGGAGGCCAACACTGAGCTGGAGGTGAAGATCCGTGACTGGTACCAGAGGCAGGCCCCGGGGCCCGCCCGTGACTACAGCCAGT  400
Moderate Psoriatic KRT17 301   AAGGTGCGTGCCCTGGAGGAGGCCAACACTGAGCTGGAGGTGAAGATCCGTGACTGGTACCAGAGGCAGGCCCCGGGGCCCGCCCGTGACTACAGCCAGT  400

KRT 17                   401   ACTACAGGACAATTGAGGAGCTGCAGAACAAGATCCTCACAGCCACCGTGGACAATGCCAACATCCTGCTACAGATTGACAATGCCCGTCTGGCTGCTGA  500
Moderate Psoriatic KRT17 401   ACTACAGGACAATTGAGGAGCTGCAGAACAAGATCCTCACAGCCACCGTGGACAATGCCAACATCCTGCTACAGATTGACAATGCCCGTCTGGCTGCTGA  500

KRT 17                   501   TGACTTCCGCACCAAGTTTGAGACAGAGCAGGCCCTGCGCCTGAGTGTGGAGGCCGACATCAATGGCCTGCGCAGGGTGCTGGATGAGCTGACCCTGGCC  600
Moderate Psoriatic KRT17 501   TGTCTTCCGCACCAAGTTTGAGACAGAGCAGGCCCTGCGCCTGAGTGTGGAGGCCGACATCAATGGCCTGCGCAGGGTGCTGGATGAGCTGACCCTGGCC  600

KRT 17                   601   AGAGCCGACCTGGAGATGCAGATTGAGAACCTCAAGGAGGAGCTGGCCTACCTGAAGAAGAACCACGAGGAGGAGATGAACGCCCTGCGAGGCCAGGTGG  700
Moderate Psoriatic KRT17 601   AGAGCCGACCTGGAGATGCAGATTGAGAACCTCAAGGAGGAGCTGGCCTACCTGAAGAAGATCCACGAGGAGGAGATGAACCCCCTGCGAGGCCAGGTGG  700

KRT 17                   701   GTGGTGAGATCAATGTGGAGATGGACGCTGCCCCAGGCGTGGACCTGAGCCGCATCCTCAACGAGATGCGTGACCAGTATGAGAAGATGGCAGAGAAGAA  800
Moderate Psoriatic KRT17 701   GTGGTGAGATCAATGTGGAGATGGACGCTGCCCCAGGCGTGGACCTGAGCCGCATCCTCAACGAGATGCGTGACCAGTATGAGAAGATGGCAGAGAAGAA  800

KRT 17                   801   CCGCAAGGATGCCGAGGATTGGTTCTTCAGCAAGACAGAGGAACTGAACCGCGAGGTGGCCACCAACAGTGAGCTGGTGCAGAGTGGCAAGAGTGAGATC  900
Moderate Psoriatic KRT17 801   CCGCAAGGATGCCGAGGATTGGTTCTTCAGCAAGACAGAGGAACTGAACCGCGAGGTGGCCACCAACAGTGAGCTGGTGCAGAGTGGCAAGAGTGAGATC  900

KRT 17                   901   TCGGAGCTCCGGCGCACCATGCAGGCCTTGGAGATAGAGCTGCAGTCCCAGCTCAGCATGAAAGCATCCCTGGAGGGCAACCTGGCGGAGACAGAGAACC  1000
Moderate Psoriatic KRT17 901   TCGGAGTTCCGGCGCACCATGCAGGCCTTGGAGATAGAGCTGCAGTCCCAGCTCAGCATGAAAGCATCCCTGGAGGGCAACCTGGTGGAGACAGAGAACC  1000

KRT 17                   1001  GCTACTGCGTGCAGCTGTCCCAGATCCAGGGGCTGATTGGCAGCGTGGAGGAGCAGCTGGCCCAGCTTCGCTGCGAGATGGAGCAGCAGAACCAGGAATA  1100
Moderate Psoriatic KRT17 1001  GCTACTGCGTGCAGCTGTCCCAGATCCAGGGGCTGATTGGCAGCGTGGAGGAGCAGCTGGCCCAGCTTCTCTGCGAGATGGAGCAGCAGAACCAGGAATA  1100

KRT 17                   1101  CAAAATCCTGCTGGATGTGAAGACGCGGCTGGAGCAGGAGATTGCCACCTACCGCCGCCTGCTGGAGGGAGAGGATGCCCACCTGACTCAGTACAAGAAA  1200
Moderate Psoriatic KRT17 1101  CAAAATCCTGCTGGATGTGAAGACGCGGCTGGAGCAGGAGATTTCCACCTACCGCCGCCTGCTGGAGGGAGAGGATGCCCACCTGACTCAGTACAAGAAA  1200

KRT 17                   1201  GAACCGGTGACCACCCGTCAGGTGCGTACCATTGTGGAAGAGGTCCAGGATGGCAAGGTCATCTCCTCCCGCGAGCAGGTCCACCAGACCACCCGCTGA  1299
Moderate Psoriatic KRT17 1201  GAACCGGTGCCCACCCGTCAGGTGCGTACCATTGTGGAAGAGGTCCAGGATGGCAAGGTCATCTCCTCCCGCGAGCAGGTCCACCAGACCACCCGCTGA  1299
```
